# Repetitive and compulsive behavior after Early-Life-Pain associated with reduced long-chain sphingolipid species

**DOI:** 10.1186/s13578-023-01106-3

**Published:** 2023-08-27

**Authors:** Alexandra Vogel, Timo Ueberbach, Annett Wilken-Schmitz, Lisa Hahnefeld, Luisa Franck, Marc-Philipp Weyer, Tassilo Jungenitz, Tobias Schmid, Giulia Buchmann, Florian Freudenberg, Ralf P. Brandes, Robert Gurke, Stephan W. Schwarzacher, Gerd Geisslinger, Thomas Mittmann, Irmgard Tegeder

**Affiliations:** 1 Institute of Clinical Pharmacology, Faculty of Medicine, Goethe-University, Frankfurt, Germany; 2 Institute of Physiology, University Medical Center of the Johannes Gutenberg University, Mainz, Germany; 3 Fraunhofer Institute for Translational Medicine and Pharmacology ITMP, 60596 Frankfurt, Germany; 4 Fraunhofer Cluster of Excellence for Immune Mediated Diseases (CIMD), 60596 Frankfurt, Germany; 5 Institute of Clinical Neuroanatomy, Neuroscience Center, Goethe University, Frankfurt, Germany; 6 Institute of Biochemistry I, Faculty of Medicine, Goethe-University, Frankfurt, Germany; 7 Partner Site Frankfurt, German Cancer Consortium (DKTK), Frankfurt, Germany; 8 Institute of Cardiovascular Physiology, Faculty of Medicine, Goethe-University, Frankfurt, Germany; 9 Department of Psychiatry, Psychosomatic Medicine and Psychotherapy, Goethe-University Hospital, Frankfurt, Germany

**Keywords:** Nociception, Cortical excitability, Multichannel electrode arrays, Compulsive behavior, Repetitiveness, IntelliCage, Calcium, Optogenetic

## Abstract

**Background:**

Pain in early life may impact on development and risk of chronic pain. We developed an optogenetic Cre/loxP mouse model of “early-life-pain” (ELP) using mice with transgenic expression of channelrhodopsin-2 (ChR2) under control of the *Advillin* (*Avil*) promoter, which drives expression of transgenes predominantly in isolectin B4 positive non-peptidergic nociceptors in postnatal mice. Avil-ChR2 (Cre +) and ChR2-flfl control mice were exposed to blue light in a chamber once daily from P1-P5 together with their Cre-negative mother.

**Results:**

ELP caused cortical hyperexcitability at P8-9 as assessed via multi-electrode array recordings that coincided with reduced expression of synaptic genes (RNAseq) including *Grin2b*, neurexins, *piccolo* and voltage gated calcium and sodium channels. Young adult (8–16 wks) Avil-ChR2 mice presented with nociceptive hypersensitivity upon heat or mechanical stimulation, which did not resolve up until one year of age. The persistent hypersensitivy to nociceptive stimuli was reflected by increased calcium fluxes in primary sensory neurons of aged mice (1 year) upon capsaicin stimulation. Avil-ChR2 mice behaved like controls in maze tests of anxiety, social interaction, and spatial memory but IntelliCage behavioral studies revealed repetitive nosepokes and corner visits and compulsive lickings. Compulsiveness at the behavioral level was associated with a reduction of sphingomyelin species in brain and plasma lipidomic studies. Behavioral studies were done with female mice.

**Conclusion:**

The results suggest that ELP may predispose to chronic “pain” and compulsive psychopathology in part mediated by alterations of sphingolipid metabolism, which have been previously described in the context of addiction and psychiatric diseases.

**Graphical Abstract:**

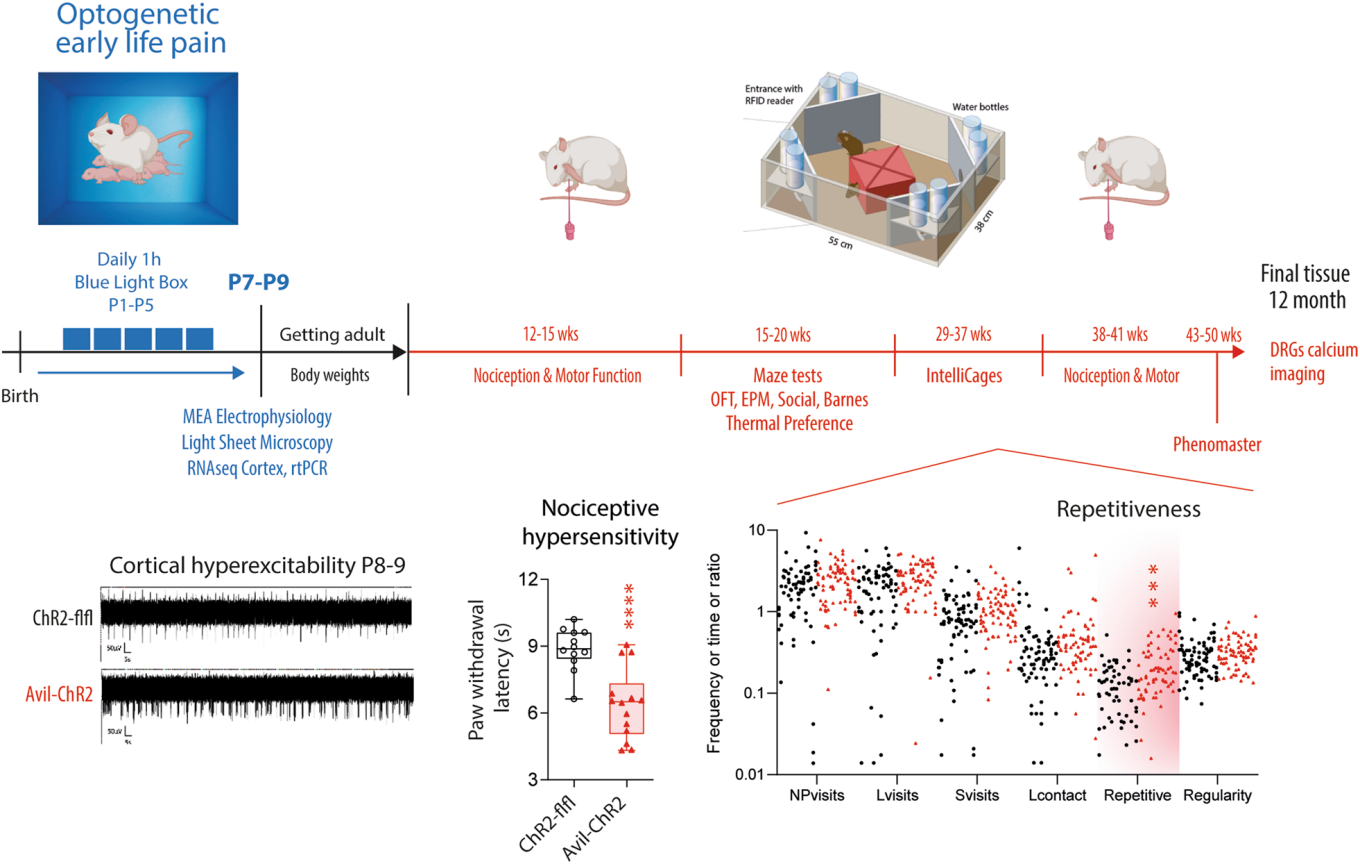

**Supplementary Information:**

The online version contains supplementary material available at 10.1186/s13578-023-01106-3.

## Introduction

Newborn and particularly preterm children are frequently exposed to various painful stimuli such as injections or venipuncture during hospitalization in the early days of life [1]. It is well accepted that pain perception and discrimination of painful stimuli starts long before birth around pregnancy weeks 20–25 [2–4] and early pain may affect cortical development and the risk of chronic pain in adult life [5–7]. The connectivity of cortical neuronal networks proceeds post-birth and is critically modulated by sensory input [8]. In mice, pleasant touch stimuli or whisker stimulation during postnatal day P1-P7 lead to apoptosis of non-used neurons and shaping of cortical networks [9–11]. The effect of nociceptive stimuli is less well understood, and mostly studied via evoked potentials and electroencephalogram recordings in human infants [12–14] and electrophysiology studies in neonatal rats [15–17]. Biological and mechanistic insights have been obtained with pinprick, skin incision or nerve injury based “early-life-pain” (ELP) rat models [6, 18–20]. Some studies suggested that nociceptive receptive fields and nociceptive sensitivity increased and remained elevated in adult life. This insight relies on injury models and is in part owing to long lasting immune activation [7, 21]. Results of non-injury pinprick are controversial [22], and interpretation of long-term outcomes is complicated because of the unknown influence of tissue injury per se, impact of rodent handling and temporary separations from siblings and mother. Even short lasting maternal deprivation impacts on the development of the endocannabinoid and dopamine system [23, 24] which may increase the risk of psychopathology, substance abuse [25, 26] and chronic pain [27, 28].

We have previously described an optogenetic Cre/loxP-mediated mouse model [29] where transgenic expression of blue light sensitive anion channel, channelrhodopsin-2 (ChR2), is mediated by Cre-recombinase that excises a STOP codon upstream of a ChR2-tdTomato fusion protein (Ai27) [30]. Cre expression was under control of the promotor for *advillin* (*Avil*), a gene that is expressed in peripheral sensory neurons [31–33]. In the dorsal root ganglia (DRGs) of postnatal mice, advillin is enriched in isolectin B4 (IB4) non-peptidergic nociceptors [34, 35]. Advillin-mediated reporter expression was also found in the autonomous nervous system, but during development and the early postnatal period up until P7 advillin expression is absent from autonomous ganglia [34]. Previously, we have demonstrated that adult Avil-ChR2 mice actively avoid blue light in a two-choice blue/red light chamber, and they withdraw the paw upon stimulation of the hind paw with a blue light-emitting-diode at high intensity (LED) [29]. The withdrawal responses or avoidance behavior were therefore interpreted as nociceptive responses, which agrees with other studies using optogenetic transgenic mice, where channelrhodopsin was directed to peripheral nociceptive neurons via Nav1.8-Cre [36], also known as SNS-Cre [37] (encoded by *Scn10a*), or via transient receptor potential, TRPV1-Cre [38].

We have now used Avil-ChR2 mice to develop an optogenetic ELP model in which litters were exposed from P1-P5 to blue light in a chamber as a group together with their Cre-negative mothers, who were not blue-light sensitive. Blue light stimulation was supposed to activate predominantly non-peptidergic nociceptors in a subtle non-harmful transdermal way. Female mice were observed up to one year of age in multiple behavioral studies including nociception, classical mazes and the IntelliCage. The early impact of ELP was studied by cortical multi-electrode array (MEA) chip electrophysiology, transcriptomics and histology, whereas persistent peripheral sensitization was revealed via calcium imaging of primary sensory neurons. Subtle alterations of brain metabolism were revealed via brain and plasma lipidomic studies addressing the concept that sphingomyelin dysmetabolism is crucially involved in psychiatric diseases [39–42] that may develop as sequelae of ELP [43].

## Methods

### Mice

Heterozygous floxed mice carrying a modified channelrhodopsin-2/td-Tomato fusion were purchased from the Jackson Laboratories (Strain #: 012567; RRID: IMSR_JAX:012567). These B6.Cg-Gt(ROSA)26Sor^tm27.1(CAG−COP4*H134R/tdTomato)Hze/J^ mice with the common name Ai27D carry a loxP flanked STOP codon in front of an improved ChR2/td-Tomato fusion protein [30]. The construct is inserted into the Rosa26 locus. Following exposure to Cre-recombinase, ChR2/td-Tomato is expressed in Cre + cells leading to blue light sensitivity. These mice can be used in optogenetic studies for rapid in vivo activation of excitable cells by illumination with blue light (450–490 nm). Floxed control ChR2/td-Tomato-flfl mice (referred to as ChR2-flfl) were crossed with male Advillin-Cre mice (Avil-Cre) [31] to cut out the STOP codon and create a ChR2/td-Tomato transgenic mouse (referred to as Avil-ChR2). The *advillin* promotor is active around birth. In postnatal mice, advillin is enriched in IB4 positive non-peptidergic nociceptors in the DRGs [34]. Advillin expression and Avil-EGFP driven reporter expression was also found in autonomic nerves and ganglia, starting to emerge beyond P7 [34]. During the first week of life, blue light sensitivity was supposed to be confined mostly to peripheral IB4 positive nociceptors. The lines have a C57BL6 genetic background. Genotyping was done by PCR with ear punches using the KAPA mouse genotyping kit (Merck), following the protocol provided by Jackson Labs (https://www.jax.org/Protocol?stockNumber=012567&protocolID=29436). Cre-mediated expression of ChR2 was assessed at the RNA and protein level. Genotyping for Cre was done as described [44] using the Primers CreA 5'-gaa agc agc cat gtc caa ttt act gac cgt ac-3' and CreB 5' gcg cgc ctg aag ata tag aag a-3'. The sample sizes for experiments at P7 (microscopy, neurogenesis, electrophysiology) were 8–10 mixed male and female mice per genotype. Nociception in young adult mice and IntelliCage observations included 15–16 female mice per group, nociception and behavioral observations in mazes in aged mice included 12 and 14 for ChR2-flfl and Avil-ChR2 female mice, respectively. For long-term behavioral studies including IntelliCages, only female mice were used to avoid group aggression and fighting. The numbers per experiment are shown in the figure legends.

### Early life pain

Male Avil-ChR2 mice (Cre + , blue light sensitive) were bred with female ChR2-flfl (Cre − , blue light insensitive) mice to generate offspring with 1:1 ration of Cre + and Cre − so that 50% of pups expressed ChR2 and were blue light sensitive. The breeding cage was kept in a Scantainer in the vicinity of a custom made Red/Blue box [29] to avoid transport. Mice received sunflower seeds for well-being. After birth, the mice were exposed daily for 1h to blue light in a Red/Blue box together with their Cre − mother from postnatal day P1 through P5. The red chamber was turned off throughout. The chamber was kept warm from outside, and pieces of their bedding material increased the comfort. Mice were euthanized at P7-9 for analysis of gene expression, cortical MEA chip electrophysiology, neurogenesis and light sheet microscopy, or mice were weaned at P21 and allowed to grow up to adult life for analysis of nociception and behavior, final calcium imaging of primary sensory neurons, and final brain and plasma lipidomic studies. Experiments were performed with Cre − litter mates as controls. The sample sizes depended on the experiments and readout and comprised 6–16 mice per genotype as outlined in the figure legends. For behavioral tests, mice were allowed to acclimatize to the experiment rooms, cages or mazes before starting experiments. Mice had free access to food and water and were maintained in climate-controlled rooms at a 12 h light–dark cycle.

The experiments were approved by the local Ethics Committee for Animal Research (Darmstadt, Germany) (V54 19c 20/15 FK1110) and the Landesuntersuchungsamt Rheinland-Pfalz (for electrophysiology). The experiments adhered to the European and GV-SOLAS guidelines for animal welfare in science and agreed with the ARRIVE guidelines.

### Red/blue box

The arena consists of a plexiglass chamber (25 × 10 × 15 cm), which can be separated by a divider into optically distinct red and blue parts by illumination from below with red (625 nm—innocuous) and blue light (460 nm—stimulation) with LEDs. To ensure homogenous illumination of the floor the horizontal LED beam is reflected by tilted mirrors, which are mounted underneath the floor. The LED radiant flux was adjusted to achieve high blue luminous intensity. The red side remained turned off throughout. During blue light exposure the behavior was monitored by the observer to ensure well-being of the pups and mother during the exposure. After the exposure, pups and mothers were closely monitored to ensure that all pups were kept close to the mother and were suckling.

### Multi-electrode- array recordings

Animals were deeply anaesthetized with 4% isoflurane and decapitated. Brains were quickly removed and transferred to 4 °C cold choline-based artificial cerebrospinal fluid (aCSF) containing 87 mM NaCl, 37.5 mM choline chloride, 25 mM NaHCO_3_, 2.5 mM KCl, 1.25 mM NaH_2_PO_4_, 0.5 mM CaCl_2_, 7 mM MgCl_2_, 25 mM glucose, oxygenated with carbogen (95% O_2_, 5% CO_2_), pH = 7.4. Next, the brain was cut into 400 µm thick coronal slices using a vibratome (Leica VT-1200-S, Leica Mikrosysteme, Wetzlar, Germany). Slices containing the somatosensory cortex were placed in the choline-based aCSF for 20 min at 37 °C, before they were recovered and incubated for another 40 min in standard aCSF (containing 125 mM NaCl, 25 mM NaHCO_3_, 1.25 mM NaH_2_PO_4_, 2.5 mM KCl, 2 mM CaCl_2_, 1 mM MgCl_2_ and 25 mM glucose, oxygenated with carbogen (95% O_2_, 5% CO_2_), pH = 7.4). Spontaneous neuronal network activity in the acute slices was recorded by a MEA system consisting of two recording chambers (MEA2100 System, Multi-Channel Systems MCS GmbH, Kusterdingen, Germany). Each MEA chip had 60 electrodes (60MEA200/30iR; Multi Channel Systems MCS GmbH, Kusterdingen, Germany) with a diameter of 30 μm and an interelectrode distance of 200 μm. The cortical slices were placed on the MEA aligning the outer cortical border along the first row of electrodes. Electrode rows number 2 and 3 corresponded to cortical layers 2/3 of the somatosensory cortex and were used for further analysis. Each MEA chip was used for multiple recordings of several brain slices in randomized order of the pups and without knowledge of the genotype. Genotyping was done post-MEA with ear punches obtained during preparation of the acute brain slices. Cortical slices were incubated for 30 min on the chip and constantly perfused at 32 °C with oxygenated (95% O_2_ and 5% CO_2_) aCSF. Spontaneous multi-unit activity (MUA) was recorded with the Multi-Channel Experimenter 2.18 (Multi Channel Systems MCS GmbH, Kusterdingen, Germany) using a 50 kHz sampling rate and a Butterworth highpass second-order filter with 200 Hz cutoff. Events bigger than the fivefold standard deviation of the noise were collected in 5-min traces using the Multi-Channel Analyzer 2.18 (Multi Channel Systems MCS GmbH, Kusterdingen, Germany). Channels were considered inactive if less than 100 spikes were detected over the recording period of 5 min. Active channels were used for further analyses.

### RNA extraction and RT-PCR

Total RNA was extracted with Qiagen RNeasy spin columns and quantified on a NanoDrop^®^ using A260/A280 and A260/A230 ratios. RNA was reverse transcribed with Thermo Scientific Verso first strand cDNA synthesis kit using oligo dT primers. QRT-PCR was performed on a TaqMan instrument with QuantStudio 5 Software (Thermo Fisher Scientific, Germany, 384 block), SYBR Green detection, and primer sets designed with Primer 3.

### RNA sequencing and analysis

P7 pups were deeply anaesthetized with an isoflurane overdose and decapitated. Cortices were rapidly removed and flash-frozen in liquid nitrogen. Total RNA was harvested using Qiagen RNeasy mini spin columns. Illumina TruSeq stranded mRNA Sample Prep Kit was used with 1 µg of total RNA for the construction of sequencing libraries. Libraries were prepared according to Illumina’s instructions. Sequencing was performed with an Illumina Next Generation sequencing system with a sequencing depth of 75 cycles.

Sample quality was assessed with demultiplexed fastq.gz files and subsequently the alignment was performed with SeqMan NGen 17 (Lasergene) using the reference genome mm10 provided from UCSC (GRCm38) as template, a minimum read length of 50 bp and automatic adapter trimming. Results were displayed with ArrayStar 17 (Lasergene) including the number of mapped reads, target length, source length and position, strand, gene names and gene IDs, annotated according to the mm10 assembly. Reads were normalized as TMM (Trimmed Means of M values) using the EdgeR package [45, 46]. Normalized reads were analyzed with ArrayStar 17. Genes were filtered for at least 12 valid values (log2 >  − 5) out of 16 biological samples, to exclude low expression genes. Data were log2 transformed, single missing values were imputed from the normal distribution, and results were displayed as scatter plots, MA-plots and Volcano plots. The P value was set at 0.05 and adjusted according to the False Discovery Rate (FDR). Hierarchical clustering was employed to assess gene expression patterns using Euclidean distance metrics. Results were displayed as heat maps with dendrograms.

Key regulated genes (based on P-value, fold change and abundance) were further analyzed for gene ontology annotation enrichments using the Gene Ontology enRIchment anaLysis and visuaLizAtion tool (GORILLA) (http://cbl-gorilla.cs.technion.ac.il/) [47]. In addition, gene set enrichment analyses (GSEA) (http://www.gsea-msigdb.org) [48] were used to assess functional implications of up- or downregulated genes and to obtain a gene ranking of the leading edge 50 up- and downregulated genes. The RNAseq data have been deposited as GEO dataset with the provisional accession number GSE200140.

### Tissue collection: brain, plasma and DRGs

Plasma and brain were dissected for lipidomics and dorsal root ganglia (DRGs) for primary neuron culture. Mice were sacrificed by carbon dioxide and cardiac puncture whereby blood was collected into K^+^ EDTA tubes, centrifuged at 1300 g for 10 min and plasma transferred to a fresh tube and snap frozen in liquid nitrogen. The brain was rapidly excised, cerebellum and olfactory bulb were removed, cut sagittal, left and right half were weighed with precision scales and snap frozen in liquid nitrogen. Samples were stored at -80 °C until analysis. DRGs were collected in Hank's balanced salt solution (HBSS) with Ca^2+^/Mg^2+^.

### Lipidomic analyses of plasma and brain tissue

Lipidomics studies followed the protocols described in [49]. Brain tissue samples were homogenized in ethanol:water (1:3, v/v, 0.25 mg tissue/µl) using a Precellys 24 (Bertin Instruments, Montigny-le-Bretonneux, France) at 10 °C. After 1:10 dilution with ethanol water (1:3, v/v), tissue homogenates equaling 0.5 mg of tissue were used for lipid extraction following the same protocol as for plasma samples. To 10 µl of mouse plasma, 75 µl of internal standards (IS) in methanol (List of IS in Additional file, item #5), 250 µl of methyl-tert-butyl-ether and 50 µl of 50 mM ammonium formate were added and mixed vigorously. After centrifugation (20,000 *g, 5 min, ambient temperature), the upper phase was transferred and the lower phase reextracted using 100 µl mixture of MTBE: methanol: water (10:3:2.5, v/v/v, upper phase) before drying under a gentle nitrogen stream at 45 °C and storage at − 80 °C. Prior analysis, samples were reconstituted in 100 µl of methanol. Analysis was performed on an Exploris 480 with a Vanquish horizon UHPLC system (both Thermo Fisher Scientific, Dreieich, Germany) using a Zorbax RRHD Eclipse Plus C8 1.8 µm 50 × 2.1 mm ID column (Agilent, Waldbronn, Germany) with a pre-column of the same type. For the 14 min linear gradient, mobile phases were (A) 0.1% formic acid and 10 mM ammonium formate and (B) 0.1% formic acid in acetonitrile:isopropanol (2:3, v/v). Data were acquired using XCalibur v4.4 including a full scan from 180 to 1500 m*/z* at 120,000 mass resolution each 0.6 s and data dependent MS/MS spectra at 15,000 mass resolution in between. Relative quantification of previously identified lipids was performed in TraceFinder 5.1 using a mass error of (± 3 ppm), the isotope ratio and the comparison of the MS/MS spectra, while calculating the area ratio to one internal standard per lipid class (all software Thermo Fisher Scientific, Dreieich, Germany). Internal standards are listed in Additional Methods.

### Primary DRG neuron culture

Primary neuron-enriched cultures of DRG neurons were prepared by dissecting DRGs of adult mice into HBSS (Merck), followed by digestion with 2.5 mg/ml collagenase A (Millipore) and 1 mg/ml dispase II (Invitrogen) before treatment with DNase (Sigma, 250 U per sample). Triturated cells were centrifuged through a 15% fat-free bovine serum albumin (BSA) solution, plated, and cultivated on poly-l-lysine and laminin coated cover slips in serum-free Neurobasal medium (Gibco-BRL) containing 1 × B27 supplement (Gibco), 1% penicillin/streptomycin (Sigma Aldrich), 200 ng/ml nerve growth factor (Gibco) and 2 mM l-glutamine (Gibco) at 37 °C and 5% CO_2_ and 95% humidity. Primary DRG neurons were used for calcium imaging,

### Calcium imaging in primary DRG neurons

Calcium fluxes were measured fluorometrically as the ratio of the absorbances at 340 and 380 nm (F 340/380) in cultured adult DRG neurons using a Leica calcium-imaging setup, with Leica DMI 4000 b inverted microscope equipped with a DFC360 FX (CCD) camera, Fura-2 filters, and an N-Plan 10x/0.25 Ph1 objective lens. Cells were loaded with 5 μM of the Ca^2+^-sensitive fluorescent dye Fura-2-AM-ester (Biotium), incubated for 40 min at 37 °C and washed three times with Ringer solution (Fresenius). Coverslips were then transferred to a perfusion chamber and were perfused with Ringer solution with a flow rate of 1–2 ml/min at room temperature. Images were captured every two seconds and were processed with the LAS AF-software (Leica). Baseline ratios were recorded for 200 s (0–200 s), before switching to 100 nM capsaicin in Ringer solution (Sigma) to activate TRPV1 ion channels for 20 s (200–220 s). After wash-out with Ringer solution, cells were perfused with 50 mM KCl (high K^+^) for 45 s (780–825 s) to assess depolarization-evoked calcium currents and the viability of the neurons. A total of 812 and 597 neurons of each four ChR2-flfl and Avil-ChR2 mice were analyzed. Data are presented as changes in fluorescence ratios (F340/380) normalized to baseline ratios. The maximum, the time of maximum and area of the fold increase versus time curve were calculated by integration. The maxima and areas were used for statistical comparisons.

### Immunofluorescence studies

Mice were terminally anesthetized with pentobarbital and transcardially perfused with cold 0.9% NaCl followed by 2.25% paraformaldehyde (PFA) for fixation. Tissues were excised, postfixed in 2.25% PFA for 2 h, cryoprotected overnight in 20% sucrose at 4 °C, embedded in small tissue molds in cryo-medium and cut on a cryotome (10 or 12 μm) or vibratome (50 µm). Slides were air-dried and stored at − 80 °C.

For analysis of neurogenesis, mice received subcutaneous injections of 60 mg/g bromodesoxyuridine (BrdU) at postnatal day P1, P3 and P5 and were perfused 48 h later (P7) transcardially with 0.9% NaCl and PFA after a terminal overdose of pentobarbital. The brain was postfixed in PFA, cryoprotected overnight in 20% sucrose and embedded in tissue-tek cryomedium. Free-floating 50 µm sections were prepared on a vibratome and stored in cryoprotection media (30% ethylene, 25% glycerol and 0.01% NaN_3_ in 0.1 M PBS) at − 20 °C until used for immunostaining. After washing in TRIS buffered saline (TBS), sections were blocked in 0.5% Triton X-100/5% BSA/TBS at room temperature (RT) for 60 min and incubated in primary Prox1 antibody (rabbit, polyclonal, 1:1000, ReliaTech) solution 0.1% Triton X-100 and 1% BSA for 72 h. After washing, sections were incubated in secondary antibody overnight. Sections were treated with 2 M hydrochloric acid at 37 °C for 30 min and 0.1 boric acid at RT for 10 min for BrdU antigen retrieval. Blocking was performed at RT for 30 min in 0.5% Triton X-100/5% BSA/TBS. After washing, anti-BrdU (rat, polyclonal, 1:250, Abcam) was applied in 0.1% Triton X-100/1% BSA/TBS overnight, followed by washing and overnight staining with the fluorochrome-labeled secondary antibody.

For LightSheet microscopy, an iDISCO + clearing method with dichloromethane (DCM) was used. PFA-fixed P7 brains were dehydrated in graded steps of methanol (20%, 40%, 60%, 80%, 100%) and subsequently cleared overnight in DCM/MeOH 2:1 vol/vol, washed in 100% MeOH, bleached in 5% H_2_O_2_ in MeOH, rehydrated and subjected to permeabilization and immunostaining with anti-active caspase-3 antibody (rabbit, polyclonal, Cell Signal). Antibody incubations were done free-floating and shaking for 4–5 days at 4 °C. After final washes, the tissue was again dehydrated, finally incubated in 100% DCM and subsequently stored in dibenzyl-ether (DBE) for imaging.

Microscopic images were captured on a Zeiss LSM confocal microscope to assess neurogenesis via BrdU/Prox1 staining. BrdU/Prox1 images were analyzed with FIJI ImageJ using the point picker and counter. Analyses were done with four mice per group. For LightSheet microscopy, samples were scanned on an Ultramicroscope II (LaVision BioTec, Bielefeld, Germany). Pictures were taken with a Neo 5.5 (3-tap) sCOMs Camera (Andor, Mod. No.: DC-152q-C00-FI) with ImSpectorPro Software and image analysis and quantification were accomplished with Imaris software (Bitplane Version 7.6).

### Behavioral analyses

**Fig. 1:**
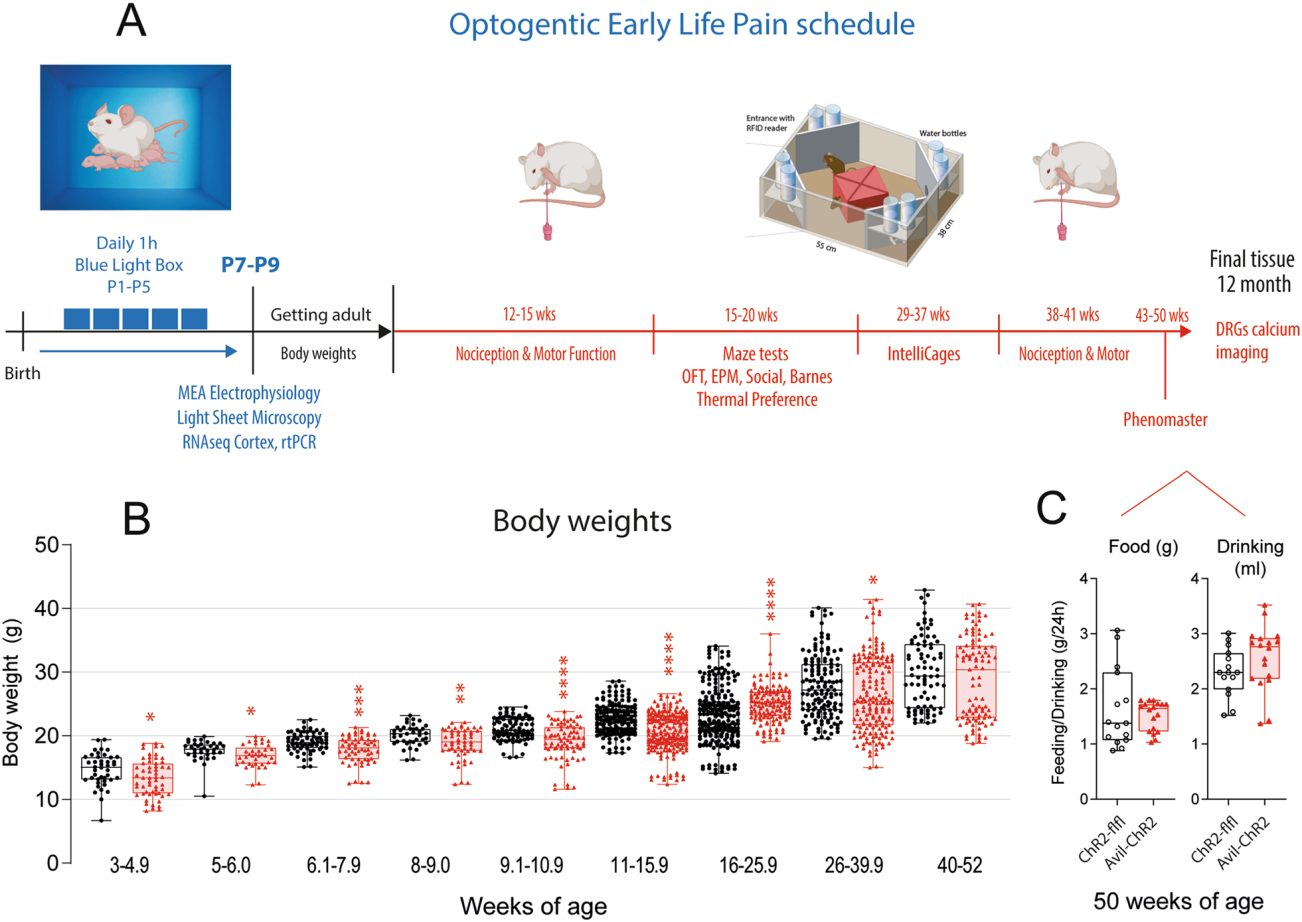
Schedule of Early-Life-Pain experiments and body weight time courses. **A** Schedule of blue light exposure of mice on postnatal day P1-P5, tissue and electrophysiology studies at P7-P8, behavior in adult mice (nociception, mazes, motor, IntelliCage) and DRG calcium imaging. **B** Time course of body weights. The scatters show body weight monitoring of n = 18 ChR2-flfl and n = 24 Avil-ChR2 female mice. The categorization into age classes considered behavioral experiments (e.g. before/after measurements) and data were analyzed via 2-way ANOVA for repeated measurements and subsequent posthoc analysis for each age class using an adjustment of alpha according to Šidák. The asterisks show adjusted P-values. *P < 0.05; **P < 0.01; ***P < 0.001; ****P < 0.0001. **C** 24 h-feeding and drinking in Phenomaster cages of ChR2-flfl and Avil-ChR2 female mice at 50 weeks of age. The weights of feeding basket and drinking bottle were controlled with precision scales and were compared with unpaired, 2-sided Student’s t-test, n = 15 ChR2-flfl and n = 18 Avil-ChR2. The box is the interquartile range, the line the median and whiskers show minimum to maximum. No difference between genotypes

Behavioural analyses were done with unbiased video-based or IntelliCage based automated observation and observer-blind measurements of paw withdrawal thresholds and rotarod running times. Mice were habituated to rooms and test chambers before baseline measurements. Experiments ending P7-P9 were done with male and female mice. Experiments performed after weaning were performed with females only, because IntelliCage experiments required female mice to avoid fighting. A summary of the schedule of tests, groups, ages, and sample sizes are presented in Fig. 1.

#### Assessment of nociception and motor function

Nociceptive and motor tests were performed at 8–12 weeks of age (n = 12 and 14) and 40–43 weeks of age (n = 15 and n = 16 for ChR2-flfl and Avil-ChR2) as described [50–53].

The latency of paw withdrawal upon mechanical stimulation was tested with a Dynamic Plantar Aesthesiometer (Ugo Basile). The steel rod was pushed against the plantar paw with ascending force (0–5 g, over 10 s, 0.2 g/s) and then maintained at 5 g until the paw was withdrawn. The paw withdrawal latency was the mean of three consecutive trials with at least 30 s intervals.

The sensitivity to painful heat stimuli was assessed as paw withdrawal latency with a Hot Plate at 52 °C, or with the Hargreaves test (IITC Life Science), where an infrared lamp was placed with a mirror system underneath the respective hind paw, and heating started by pressing the start button. The lamp emits a heat-beam until the paw is withdrawn, which stops the lamp. The mean paw withdrawal latency of three tests with at least 10 min intervals was used for statistical analysis. The test sequence of right and left paws was random.

Motor coordination and running performance were assessed at 14 and 40 weeks of age with the accelerating or constant speed rotarod (accelerating: 16–32 rpm, ramp 3 rpm/min, cut-off 5 min; Ugo Basile). Mice performed short training runs for adaptation before test measurements. The running time in three test trials was averaged. The cut-off time was 300 s for accelerating runs and 120 s for constant speed.

#### Assessment of temperature preferences on a thermal gradient ring (TGR)

A thermal gradient ring (TGR) was used to assess the temperature preferences and exploration of the ring platform that consists of a circular running ring platform that allows free choice of the comfort zone. The dimensions of inner and outer ring diameters are 45 cm and 57 cm. The inner walls consist of plexiglass and the outer walls of aluminum. Both are 12 cm high and build a 6 cm wide circular running arena. The aluminum surface provides a temperature gradient that is controlled with two Peltier elements and constantly measured with infrared cameras. The arena is divided into mirror-image semicircles of 12 temperature zones, so that duplicate readouts are provided for each zone. During measurements, the running track is illuminated, and the mouse behavior is videotaped with a regular CCD camera, mounted above the mid-point of the ring. The time spent in zones and temperature preferences are analyzed with the TGR ANY-Maze video tracking software (Stoelting).

#### Phenomaster

The TSE Phenomaster provides automated high precision monitoring of feeding, drinking and voluntary wheel running (VWR) in a home cage. Drinking and feeding behavior were monitored with high-precision weight sensors for liquid and food dispensers, which are integrated into the lid of the cage. The running wheel was freely accessible for appetitive running. Mice were adapted to the drinking bottles for one week in their home cage and to the Phenomaster cage for one day before starting the experiment. Drinking, feeding and voluntary wheel running were recorded for 24 h.

#### Open field (OFT) and elevated plus maze (EPM) and Barnes maze

Mice were placed in the middle of an open field (50 × 50 cm width, 38 cm height) and allowed to move freely for 10 min. They were observed per video camera. Virtual zones were defined as centre and border.

In the elevated plus maze (EPM) test, mice were placed in the centre of a standard EPM with two open arms and two closed arms with grey plastic walls (10 × 50 cm, height 50 cm above ground) and allowed to move freely for 10 min. In both tests, locomotion, visits to and times spent in zones were analysed with VideoMot2 which uses a 2-point tracking (TSE Systems).

The Barnes Maze protocol consisted of three phases: habituation, learning, and reversal learning. In the habituation phase, mice were set under a plastic cylinder for 30 s in the middle of the maze, and were then directed to the target hole, where they were allowed to enter the shelter within 3 min. If not, they were nudged into it and allowed to stay there for 1 min. The habituation was done for 3 days with 3–5 trials per day. In the initial learning phase (3 days, 1 trial each) mice were allowed to freely explore the maze for 5 min to find and enter the target hole. In the subsequent Reversal Learning phase (4 days, 1 trial each) the target box was moved to the opposite side of the maze. The latency to escape and distances were video recorded and analysed with EthoVision XT 11.5 software (Noldus, Wageningen, Netherlands).

#### Social cognition and memory

Social cognition and memory were tested according to standard protocols in a three-chamber box (each chamber 18.5 × 38 cm) [54]. The middle chamber was connected to the outer chambers by doors, which can be closed. A cylindrical enclosure was placed into the corners of each outer compartment. Mice were habituated to the environment before start. At the experiment day, mice were acclimatized to the middle chamber for 5 min with closed doors. The doors were then opened, and mice were allowed to explore the chambers and enclosures, one empty, the other with a stimulus mouse for 10 min (social cognition). Subsequently, a second mouse was added to the empty enclosure, again for 10 min to assess behavior towards social novelty. The trials were recorded with a video camera and analyzed with VideoMot2 software (TSE Systems).

#### IntelliCage set up and tasks

The IntelliCage (NewBehavior AG, Zurich, Switzerland) [55–57] consists of four operant corners, each with two water bottles, sensors, LEDs and doors that control the access to the water bottles. The system fits into a large cage (20 × 55 × 38 cm, Tecniplast, 2000P) and allows housing of 16 mice per cage. Four triangular red houses are placed in the center to serve as sleeping quarters and as stands to reach the food. The floor is covered with standard bedding. Mice are tagged with radio-frequency identification (RFID)-transponders, which are read with an RFID antenna integrated in the corner entrances. The corners give access to two holes with water bottles, which can be opened and closed by automated doors. Mice have to make nosepokes (NP) to open the doors for water access. The IntelliCage is controlled by a computer with IntelliCage Plus software, which executes pre-programmed experimental tasks and schedules. The numbers and duration of corner visits, NP, and licks are automatically recorded without the need for handling of the mice during the recording times.

IntelliCage tasks address several different aspects of cognition as well as circadian rhythms and social interactions and were run sequentially. The tasks followed previously established protocols [56–58]. The IntelliCage experiments were done in female mice to avoid fighting. Up to 16 mice were housed per cage (8/8 and 7/8 of each genotype). Mice were adapted to the system for 2 weeks with free access to every corner, with all doors open, and water and food ad libitum. This free adaptation (FA) was followed by 2 weeks NP adaptation during which the doors were closed, the first NP of the visit opened the door for 5 s and to drink more, the animals had to leave the corner and start a new visit. In the place preference learning (PPL) task mice had to learn to prefer a specific corner for 10 days, where they got the water reward. Each 4 mice were assigned to one corner. Only the first correct NP opened the door, and an incorrect NP had no effect. After conditioning to the corner, PPL reversal learning (PPLrev) was assessed by switching the rewarding corner to the opposite side for 10 days. Learning and memory were supported by LEDs.

### Statistics

Group data are presented as mean ± SD or median ± IQR for non-parametric data as specified in the respective figure legends. Behavioral time course data show mean ± sem. Data were analyzed with SPSS 27 and GraphPad Prism 9 and Origin Pro 2022, and MetaboAnalyst 5.0 for ANOVA-simultaneous component analysis (ASCA) and Random Forest (https://www.metaboanalyst.ca) [59]. Data were mostly normally distributed, or log-normally distributed. For testing the null-hypothesis that groups were identical, two groups were compared with 2-sided, unpaired Student’s t-tests. The Mann Whitney U test (2 groups) was used as a non-parametric alternative in case of violations of t-test requirements. Time course data were submitted to 2-way analysis of variance (ANOVA) using e.g. the factors ‘time’ and ‘genotype’. In case of significant differences, groups were mutually compared at individual time points using post hoc t-tests according to Dunnett, i.e. versus the control group, or according to Šidák. Asterisks in figures show multiplicity-adjusted P-values. ANOVA-simultaneous component analysis (ASCA) [60] was used for analysis of multiple behavioral features in sequential tasks. ASCA is a combination of ANOVA and PCA plus feature extraction method for multivariate data to model two major components and their interaction, which were “genotype” and “time/task”. The feature extraction is based on “leverage”, which is a measure of the importance of a feature’s contribution to the multivariate fitted ASCA-model, and the squared prediction error (SPE), which is an evaluation of the goodness of fit of the model to a particular feature. A Random Forest supervised learning algorithm was used assess the prediction of group membership and classification of behavioral features according to their importance. For multivariate analyses, data were normalized using Range Scaling or Auto Scaling. Specific analyses of electrophysiology, calcium fluxes and RNAseq are explained in the methods above. Volcano plots were used to assess fold differences of lipids versus the negative logarithm (Log_10_) of the ttest P value according to standard procedures. Lipidomic data were further analyzed with MetaboAnalyst 5.0 (Random Forest, Partial Least Square Discrimination Analysis, PLSDA) to assess the predictability of group membership based on brain lipids.

## Results

### Temporary lower body weights after ELP in Avil-ChR2-expressing mice

Mice were exposed to blue light from P1 to P5 (see Fig. 1A), and body weights were monitored throughout life (Fig. 1B). Body weights of Avil-ChR2 mice were lower from the time of weaning at 3–4 weeks of age up to 16 weeks. They recovered and temporarily overtook controls (16–25 weeks) and then stabilized at body weights similar to controls. There was no difference at final time points. Hence, Avil-ChR2 recovered normal body weights. Consistently, drinking and feeding behaviors were equal at 50 weeks of age as determined by Phenomaster analysis (Fig. 1C).

### Cortical hyperexcitability after ELP

**Fig. 2:**
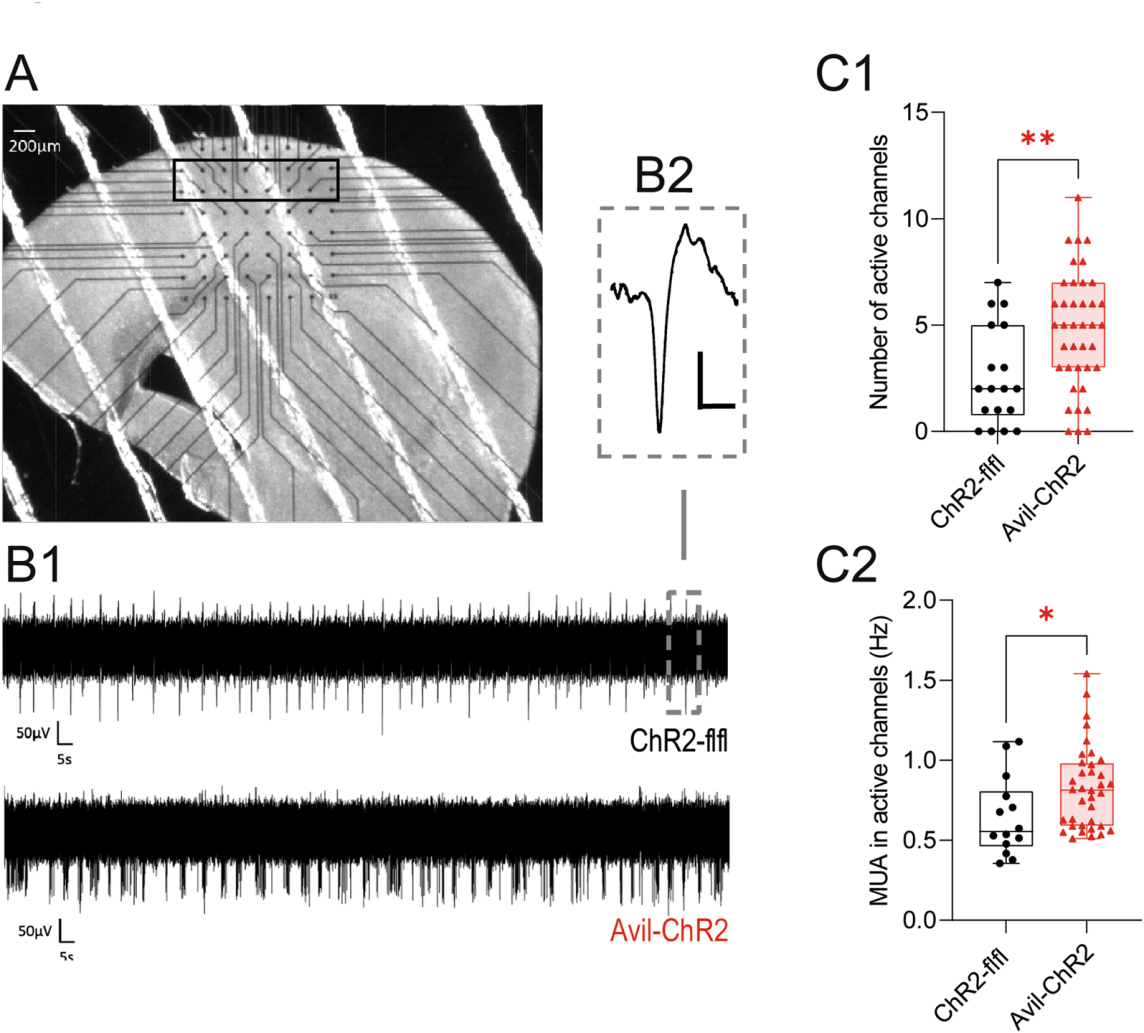
Cortical hyperexcitability in Early-Life-Pain mice at P7-8. Spontaneous cortical network activity of acute brain slices of ChR2-flfl and Avil-ChR2 mice at P8-9 after exposure to blue light on postnatal day P1-P5. **A** Photograph of an acute brain slice located on top and in touch with the 60-recording electrode MEA-chip setup. The gray square represents the recording area within the somatosensory cortex. **B**_**1**_ Representative voltage traces at one of the recording electrodes of the MEA in somatosensory cortex of ChR2-flfl (top trace) and Avil-ChR2 (lower trace). Note the higher number of spike events in the Avil-ChR2 group (lower voltage trace). **B**_**2**_ Inset showing one spike event at higher magnification (scale bar = 50 µV/1 ms). **C1**: Summary diagram showing the number of MEA electrodes for each experimental group, where multi-unit activity (MUA) could be detected in somatosensory cortex. **C2**: Summary diagram showing the frequency of the observed MUA in all active channels of ChR2-flfl and Avil-ChR2 slices (n = 10 mice per genotype). Each datapoint represents data from one brain slice. The data were statistically evaluated by an unpaired, 2-sided Student’s t-test. *P < 0.05. Transcriptomics reveal lower cortical expression of synaptic genes

The network of the somatosensory cortex was investigated by electrophysiological multi-electrode array recordings of acute brain slices at P8-P9 (Fig. 2A, B1, B2), i.e. after a stimulation-free interval of 3–4 days (blue light exposure P1-P5). Litter mates were simultaneously exposed to the blue chamber and processed randomly for electrophysiology without knowledge of the genotype. MEA recordings showed an increased number of active MEA-channels/electrodes in Avil-ChR2 mice compared to ChR2-flfl control mice (Fig. 2C1). Furthermore, the cortical activity measured by the frequency of the multi-unit activity (MUA) in the active MEA-channels was significantly higher in Avil-ChR2 mice as compared to ChR2-flfl control mice (Fig. 2C2).

**Fig. 3:**
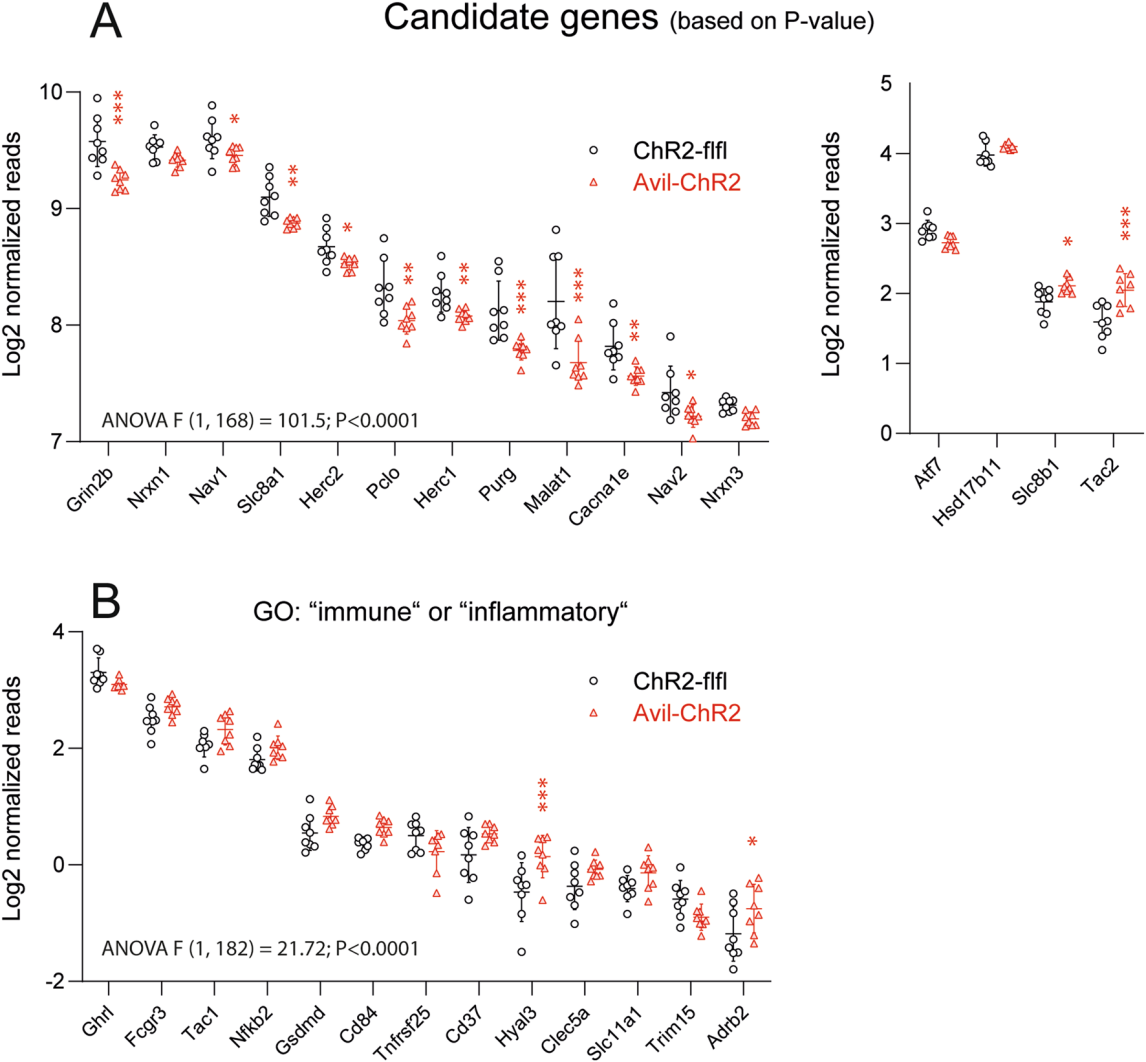
RNA sequencing of the brain cortex in Early-Life-Pain mice at P7. ChR2-flfl and Avil-ChR2 mice were exposed to blue light in a chamber on postnatal day P1-P5 together with the Cre-negative blue-insensitive mother. At P7, mice were euthanized and brain cortices subjected to transcriptome analysis via mRNA sequencing (n = 8 pups per genotype). The overview Volcano plot is shown in Additional file 1: Figure S1A. The abbreviations of the genes are the official gene symbols. Normalized counts of the presented genes along with gene description and GO terms are included as a Additional file 2: “Gene information” Excel file. RNAseq data have been deposited to the GEO database with the accession GSE200140. **A** Scatter plots of top candidate genes sorted according to gene abundance (normalized reads) and P-value. **B**: Scatter plots of genes with GO annotation “immune” or “inflammation”, P-value < 0.1 and Log2 difference < − 0.2 (down) or > 0.2 (upregulated). Low expression genes were filtered out. Each scatter is one mouse. Gene expression was compared per FDR adjusted t-test, and asterisks show the q-values. *q < 0.05, **q < 0.01, ***q < 0.001

In parallel to the MEA schedule, another group of mice was euthanized at P7 for RNAseq of brain cortices. As above, they were exposed to blue light at P1-P5. Volcano plots (Additional file 1: Figure S1A) show an overview of gene regulations. Fold changes were low (mostly within twofold range), but candidate analyses based on P-values revealed significant lower expression of genes associated with synapses in Avil-ChR2 cortices (Fig. 3A). Key downregulated hits were the glutamate receptor subunit *Grin2b* and the long non-coding RNA, *Malat1*. Further candidates were *neurexin 1* and *3* (*Nrxn1*, *Nrxn3*), *E3-ubiquitin ligase Herc1/Herc2* and *voltage gated sodium channel Nav1/2*, each represented by two isoforms, and pre-synaptic cytomatrix protein *piccolo* (*Pclo*). Tachykinins (*Tac1* and *Tac2*) encoding precursor of neurokinin-B were upregulated. The genes have all been described as candidate genes in neuropsychiatric diseases. Lower expression would agree with activity dependent synaptic refinement [61–63]. Because ELP was shown to activate the immune system, we searched for all genes with Gene Ontology terms of “immune” or “inflammatory” and subsequently filtered for P-values < 0.1 and Log2 difference < − 0.2 or > 0.2. The analysis revealed a subtle increase of pro-inflammatory markers in Avil-ChR2 cortices (Fig. 3B). There were no differences of neurogenesis based on BrdU immunofluorescence at P7 between genotypes (Additional file 1: Figure S1B), and there were also no differences of apoptosis associated genes, gross 3D brain morphology and active caspase 3 immunofluorescence (Additional file 1: Fig S1C, Additional files 4 and 5 mp4 files).

### ELP causes long-lasting nociceptive hypersensitivity in adult mice

**Fig. 4:**
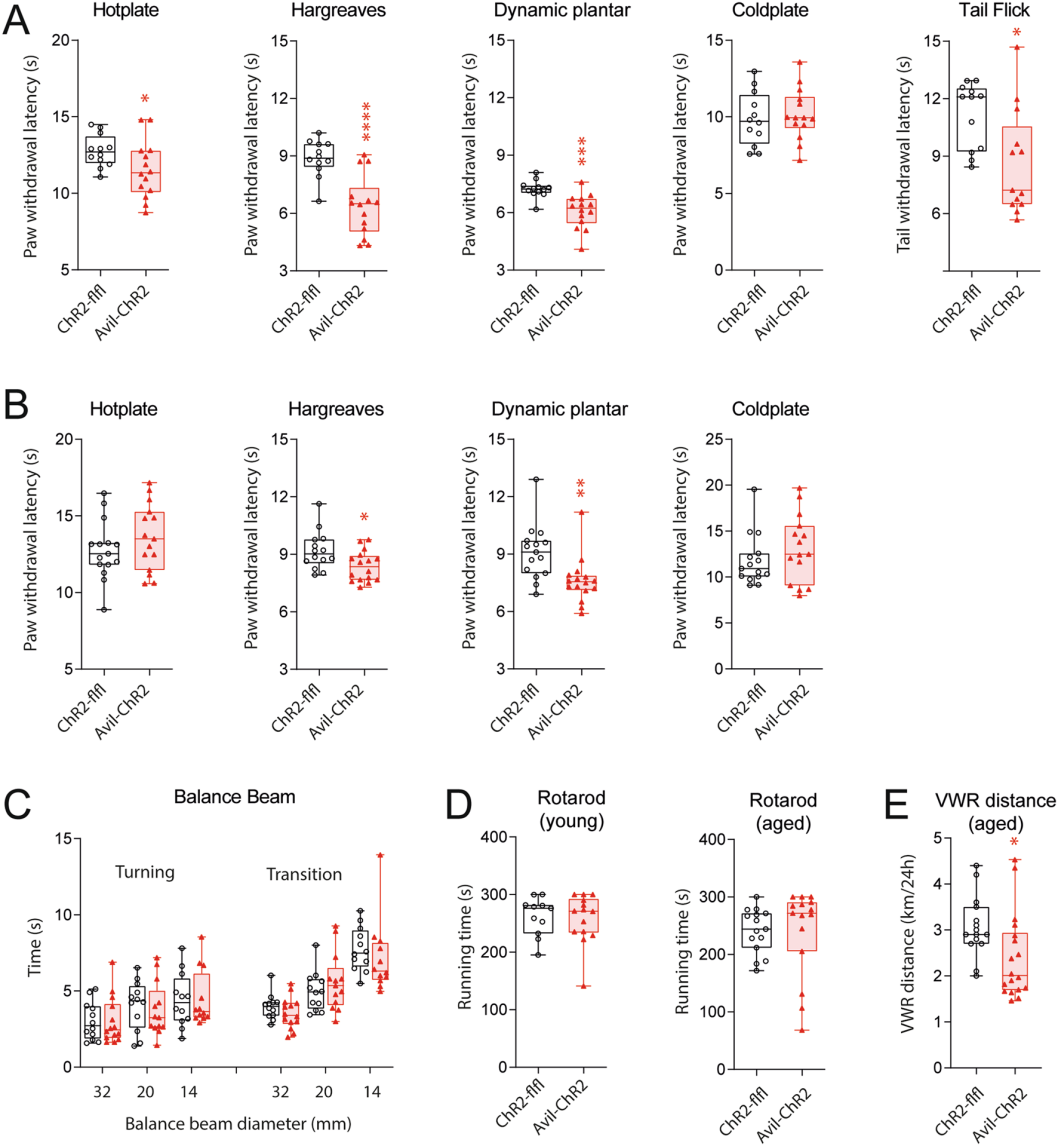
Nociceptive and motor function behavior in early and late adult life of ELP mice. ChR2-flfl and Avil-ChR2 mice were exposed to blue light in a chamber on postnatal day P1-P5 together with the Cre-negative blue-insensitive mother. Behavior was observed in adult female mice. **A** Nociceptive paw withdrawal latencies (PWL) of young adult 8–12 weeks old ChR2-flfl (n = 12) and Avil-ChR2 mice (n = 14) on heat stimulation (Hargreaves, Hotplate), mechanical stimulation (dynamic plantar test), cold stimulation (Coldplate) and heat evoked tail flick latency. Latencies were compared by unpaired, 2-tailed t-test, *P < 0.05; ***P < 0.001; ****P < 0.0001. **B** Nociceptive PWL of aged (38–40 wks) ChR2-flfl (n = 15) and Avil-ChR2 mice (n = 16). Stimulations and statistics as in A. **C** Balance beam performance at 15 weeks of age with decreasing beam diameters (32 mm, 24 mm, 16 mm). On test start mice were placed at the tip facing the open end. “Turning” is the time needed to turn around to face the home box. “Transition” is the time needed to return to the box. Data were compared by 2-way ANOVA for the within subject factor beam diameter and the between subject factor genotype. No difference between genotypes.**D** Running times (fall off latencies) on an accelerating rotarod in young adult (15 wks) and aged (40 wks) ChR2-flfl and Avil-ChR2 mice. Running times were compared via unpaired, 2-tailed t-test. No difference between genotypes.**E** 24 h-voluntary wheel running (VWR) distances of aged ChR2-flfl and Avil-ChR2 mice at 50 weeks of age in Phenomaster cages. Running distances were compared via unpaired, 2-tailed t-test; *P < 0.05. The boxes show the interquartile range, the line is the median, whiskers show minimum to maximum and scatters are individual mice

We hypothesized that early life pain (ELP) may affect pain sensitivity during adulthood, and tested nociceptive paw withdrawal latencies upon mechanical, heat and cold stimulation in adult female mice at 2–3 months and at 12 months of age. In young Avil-ChR2 mice, paw withdrawal latencies upon mechanical and heat stimulation were strongly reduced as compared with ChR2-flfl controls indicating nociceptive hypersensitivity (Fig. 4A). The difference between genotypes was fading upon aging but some nociceptive hypersensitivity was still evident at 12 months of age (Fig. 4B).

**Fig. 5:**
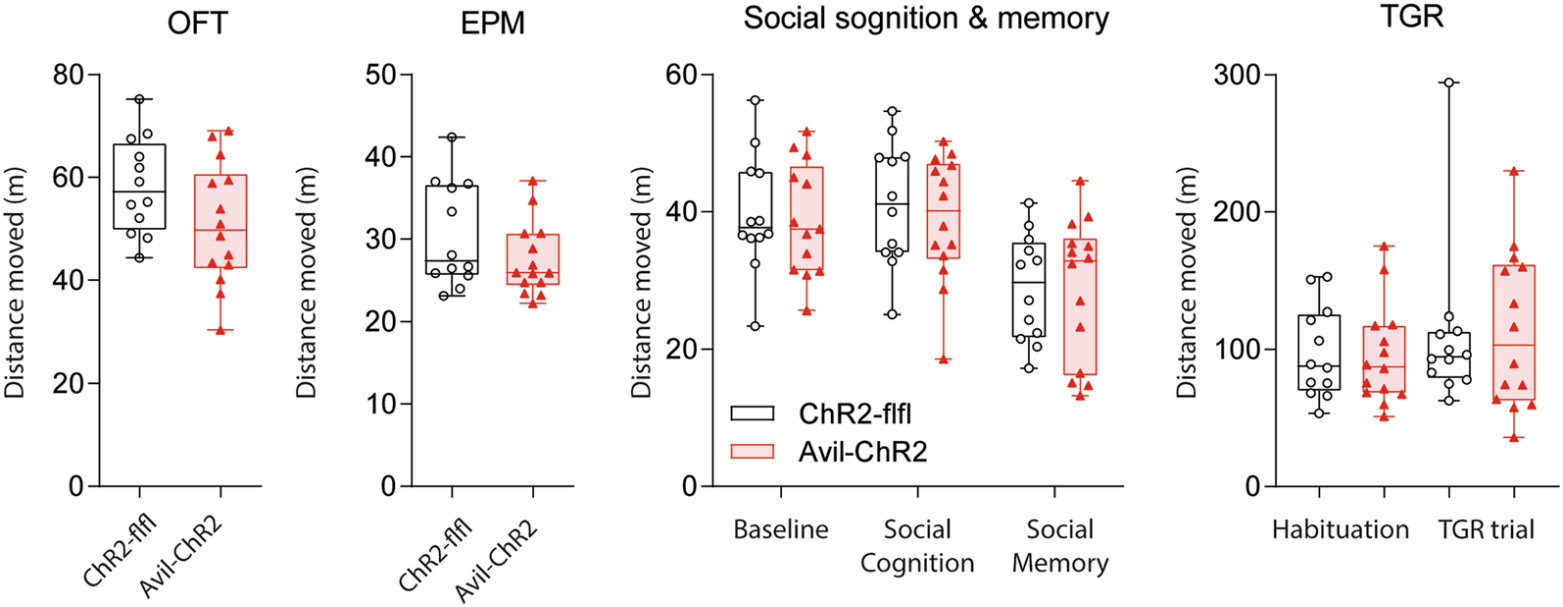
Locomotion (distance moved) in Maze tests in ELP mice. ChR2-flfl and Avil-ChR2 mice were exposed to blue light in a chamber on postnatal day P1-P5 together with the Cre-negative blue-insensitive mother. Behavior was observed in adult female mice. The box/scatter plots show distances travelled during the 10 min observation period in the Open Field Test (OFT), Elevated Plus Maze (EPM), “3-chamber, 2-phases” test of Social Cognition & Memory and during a 30 min habituation period at ambient temperature in a Thermal Gradient Ring (TGR) maze. Mice were free to move or rest in the respective maze. The travel distances are readouts for locomotion, curiosity and activity. They were compared by unpaired, 2-tailed t-tests and did not differ between genotypes. Groups comprised n = 12 for ChR2-flfl and n = 14 for Avil-ChR2

**Fig. 6:**
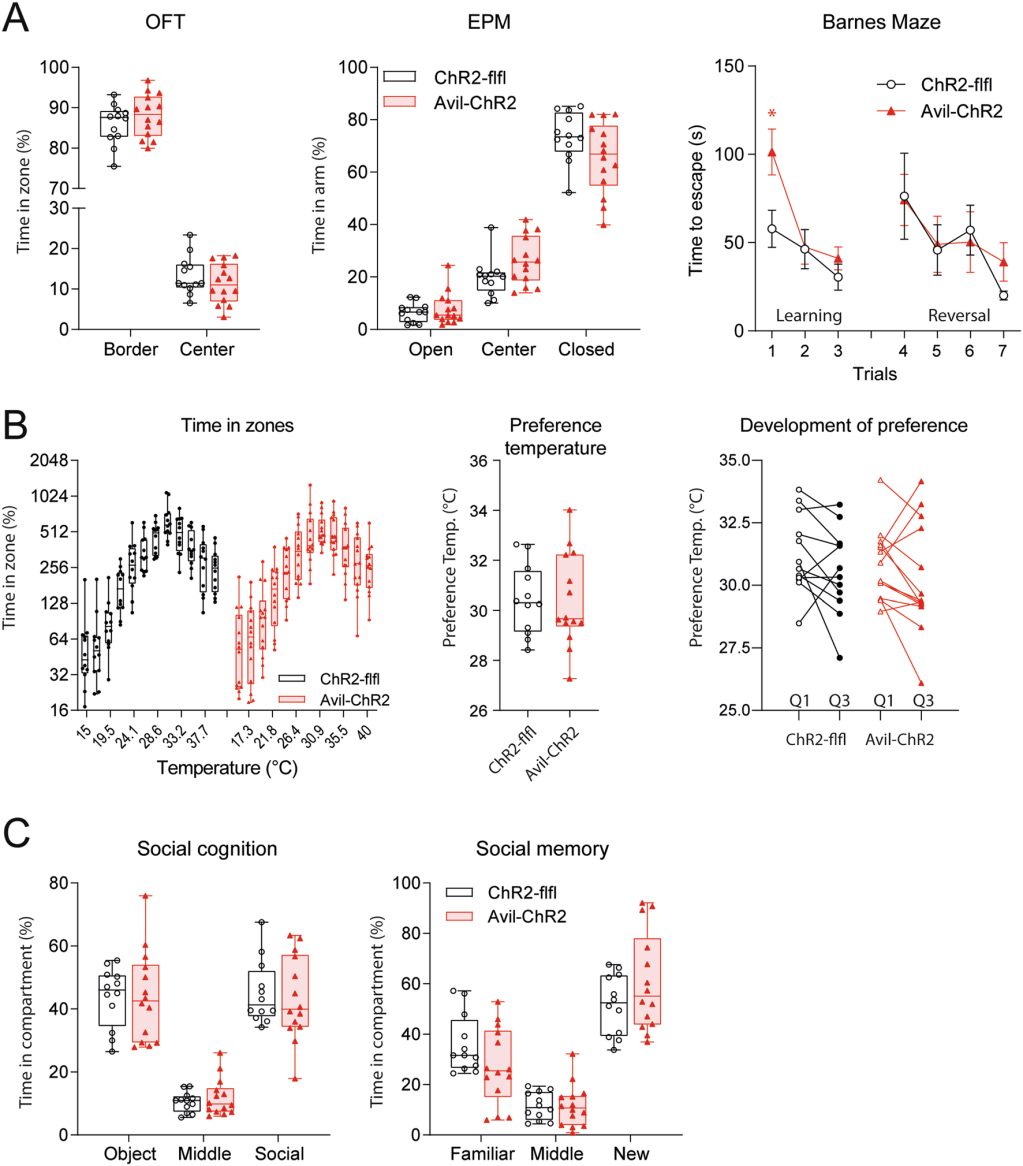
Behavior in Classical Maze tests and Thermal Gradient Ring in ELP mice. ChR2-flfl and Avil-ChR2 mice were exposed to blue light in a chamber on postnatal day P1-P5 together with the Cre-negative blue-insensitive mother. Maze tests were done in adult n = 12 and n = 14 mice at 16–20 weeks of age. **A** Behavior in open field test (OFT), elevated plus maze (EPM) and Barnes maze in ChR2-flfl (n = 12) versus Avil-ChR2 mice (n = 14). OFT box/scatter plots show the relative times spent in a virtual border zone and center zone. EPM plots show the relative times spent in open and closed arms and the transition center square. OFT and EPM are shown as percentages of the observation time, which was 10 min. Barnes maze plots show the latencies to escape in three learning and four reversal learning trials. OFT and EPM measure anxiety (border, closed) versus curiosity (center, open). The Barnes maze measures spatial learning and memory. Data were compared by 2-way ANOVA for the within subject factors “OFT zone”, “EPM arm” or “Barnes trial” and the between subject factor “genotype”. There was no difference between genotypes in OFT and EPM. For the Barnes maze, the escape latency was longer in Avil-ChR2 mice but only in the first trial (*P < 0.05). **B** Times spent in temperature zones of ChR2-flfl (n = 12) versus Avil-ChR2 mice (n = 14) at 16 weeks of age on a Thermal Gradient Ring (TGR) with a temperature gradient of 15–40 °C. The observation time was 60 min. Preference temperatures did not differ between groups. The right panel shows the change of the preference temperature in degrees Celsius from Q1 to Q3. Data were compared with 2-way ANOVA (left), unpaired (middle) and paired t-test (right). There was no difference between genotypes. **C** Behavior in a three-chamber/two-phases test of social cognition & memory (mice as in A/B). Box/scatter plots show the relative time spent in the three chambers of the box. In social cognition, one outer chamber presents a mouse, the other an object. In social memory, the outer chambers present a novel versus familiar mouse. Data were compared by 2-way ANOVA for the within subject factors “chamber” and the between subject factor “genotype”. There was no difference between genotypes. Boxes show the interquartile range, the line is the median, whiskers show minimum to maximum and the scatters represent individual mice

There was no difference in motor function tests (Fig. 4C–E) including balance beam tests of motor coordination (Fig. 4C) and Rotarod running times (Fig. 4D), but voluntary wheel running times and distances were lower in aged Avil-ChR2 mice (Fig. 4E) showing low engagement in this rewarding activity. There were no differences in walking distances in classical maze tests (Fig. 5), i.e. OFT, EPM, Social cognition & memory and TGR, which were all performed during daytime. Behavioral readouts of anxiety in OFT and EPM and learning in Barnes Maze were equal in both groups (Fig. 6A). There was also no difference between genotypes in preferences of well-being temperature in TGR (Fig. 6B), and readouts of social cognition and memory in a 3-chamber test were also equal.

### ELP causes repetitive behavior but no cognitive deficits in middle aged mice

**Fig. 7:**
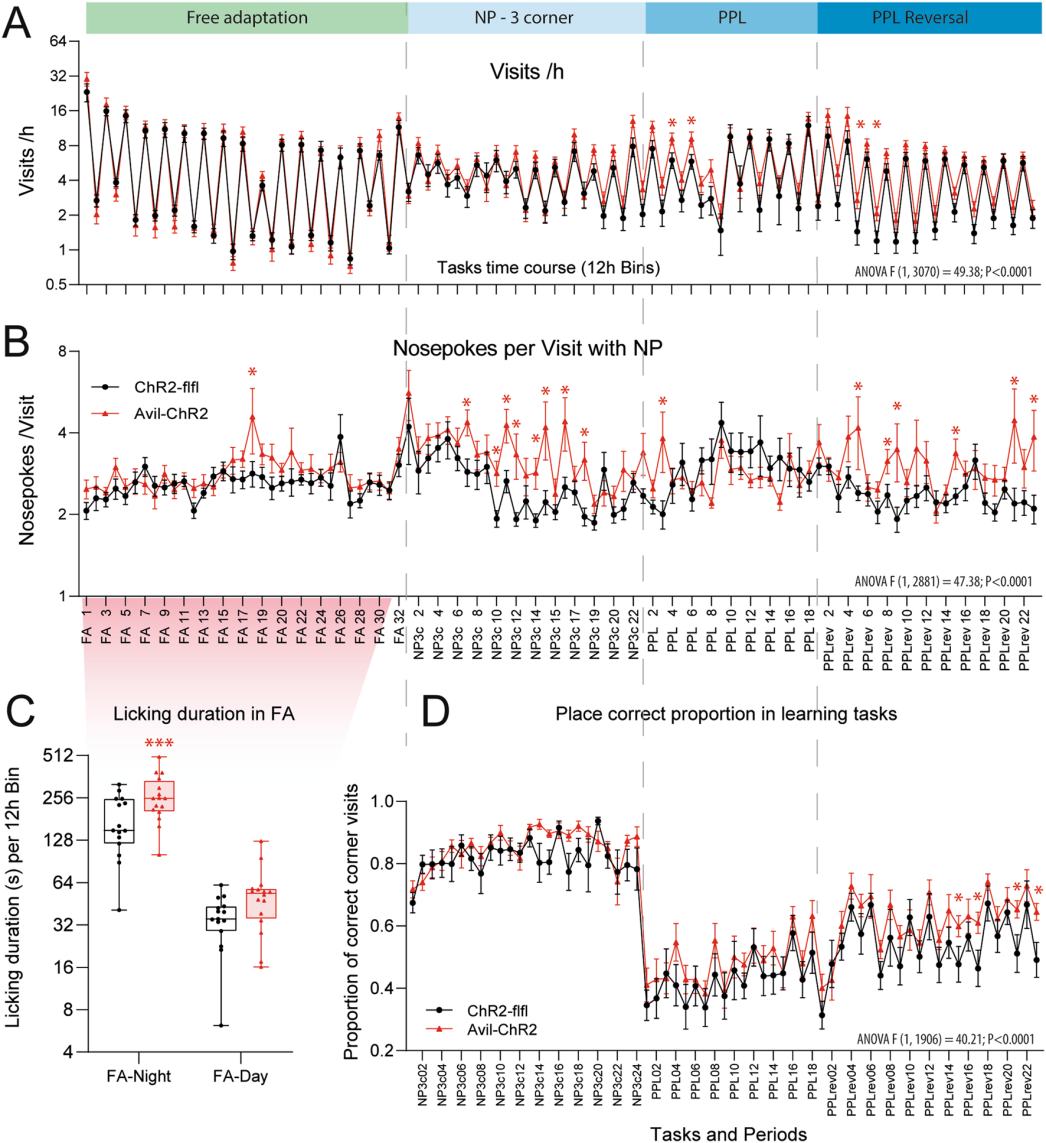
IntelliCage daytime & nighttime activity and preference learning in ELP mice. ChR2-flfl and Avil-ChR2 mice were exposed to blue light in a chamber on postnatal day P1-P5 together with the Cre-negative blue-insensitive mother. IntelliCage observations started at 30 weeks of age and lasted 9 weeks. The experiment included n = 15 ChR2-flfl and n = 16 Avil-ChR2 female mice. Mice were trained in sequential tasks of increasing difficulty. Tasks were free adaptation (FA), nosepoke adaptation (NP 3 corner, NP3c), place preference learning (PPL) and reversal place preference learning (PPL-rev). **A** Time course of the daytime and nighttime activity represented as corner visits per hour (Visits/h) in 12-h intervals (12 h Bins). The fluctuations of activity reveal the circadian rhythms. Overall corner visiting activity was similar in both genotypes except few time points in PPL and PPLrev. Actograms (Additional file 1: Fig S2) support a moderately increased activity in PPL. *P < 0.05. **B** Time course of the ratio of Nosepokes per Visit (NP /Visit) in 12 h Bins. The ratio is an individual relative stable trait influenced by exploratory drive, motivation, compulsiveness, and attention. The NP /Visit ratio was higher in Avil-ChR2 mice. *P < 0.05. **C** During Free Adaptation (FA) Avil-ChR2 mice show longer licking duration in the night (please also see Additional file 1: Fig. S3 and Additional file 4: Tables of Multivariate Statistics) which agrees with compulsive licking. Licking in FA was ad libitum, not restricted by door opening times. Comparison by 2-way ANOVA “Day time” X “genotype” and posthoc Šidák for genotype. ***P < 0.001. **D** Time course of the proportion of correct corner visits in learning tasks with either three correct corners (NP 3-corner, NP3c) or one correct corner (PPL, PPL-reversal). In PPL reversal the correct corner was switched to the opposite side as compared to PPL. *P < 0.05. Line graphs of the time courses show the mean ± sem of n = 15 ChR2-flfl and n = 16 Avil-ChR2 female mice. Time courses were compared by 2-way ANOVA for repeated measurements with the within subject factor “time” and the between subject factor “genotype”, and subsequent posthoc comparison for each time point for genotype. Asterisks show *P < 0.05 (non-adjusted 2-group comparisons)

**Fig. 8:**
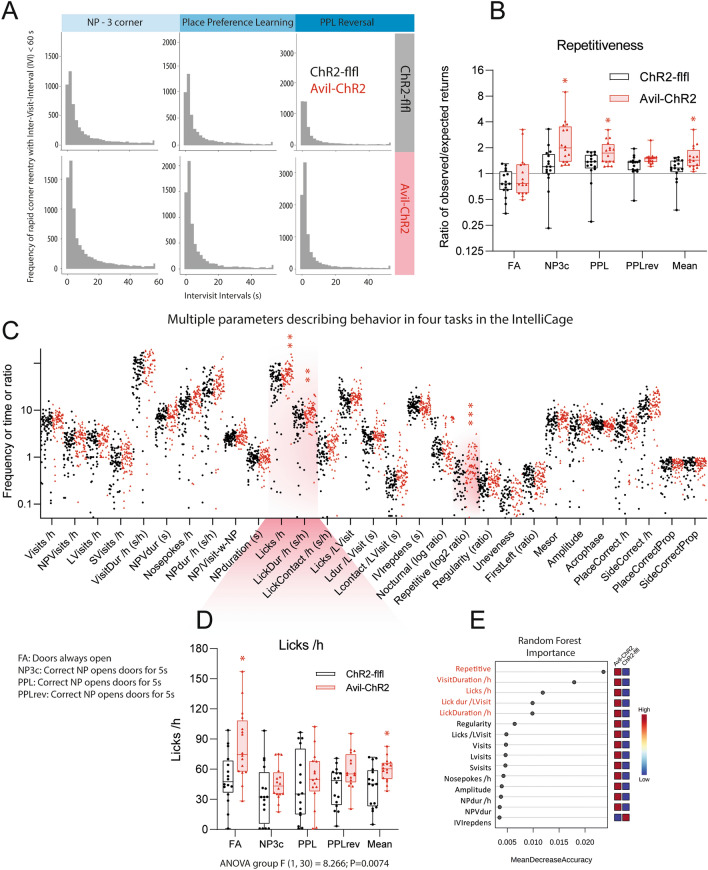
IntelliCage multivariate behavior reveals repetitiveness and compulsive licking. ChR2-flfl and Avil-ChR2 mice were exposed to blue light in a chamber on postnatal day P1-P5 together with the Cre-negative blue-insensitive mother. IntelliCage observations started at 30 weeks of age and lasted 9 weeks. The experiment included n = 15 ChR2-flfl and n = 16 Avil-ChR2 female mice. **A** Frequency of rapid corner re-entries with short Inter-Visit-Interval (IVI < 60 s) in learning tasks with either three correct corners (NP 3-corner, NP3c) or one correct corner (PPL, PPL-reversal). The frequency of short IVI's is increased in Avil-ChR2 mice suggesting repetitive behavior. **B** Repetitiveness describing the ratio of early observed versus expected returns to the same corner during different tasks (FA, NP3c, PPL, PPLrev, overall mean). The box shows the interquartile range, the line is the median, whiskers show minimum to maximum, the scatters are individual mice. Data were compared with 2-way ANOVA for the factors “Tasks” X “genotype” and posthoc comparison for “genotype” with adjustment of alpha according to Šidák.**C** Analysis of multiple dimensions of IntelliCage behavior represented by 29 different behavioral parameters. For each mouse, “per-day-values” of each parameter were averaged for the duration of each task (FA, NP3c, PPL, PPLrev) and for the toral observation time (overall mean). Hence, five mean values were obtained for each parameter for each mouse, and therefore each mouse is represented by five scatters for each parameter. Data were compared with 2-way ANOVA for the factors “IC-parameter” X “genotype” and posthoc comparison for “genotype” with adjustment of alpha according to Šidák. Avil-ChR2 mice show higher repetitiveness, as revealed by a higher frequency of fast returns to the same corner irrespective of the success in this corner. Avil-ChR2 mice also show a higher frequency of Licks /h and Licking duration /h. **D** Box/scatter plots show Licks /h during different tasks (FA, NP3c, PPL, PPLrev, overall mean). Statistics as in B. Licks were particularly high in FA where doors were open allowing licking ad libitum. During learning tasks lick duration was restricted by a door closing time of 5 s. **E** Random Forest importance of behavioral features for prediction of group membership (Please also see Additional file 1: Tables of RF statistics). *FA* free adaptation; *NP3c* Nosepoke adaptation with three correct corners; *PPL* place preference learning; *PPLrev* place preference reversal learning. Statistics: *P < 0.05, **P < 0.01; ***P < 0.001)

To further address ELP consequences of social and cognitive behavior and putative differences that manifest preferably during active times in the night we employed IntelliCages, which allow for around the clock monitoring of corner visits, nosepokes, and licks and several parameters that are deduced from frequencies, intervals, duration, preferences, circadian rhythms and sequences (Figs. 7, 8, Additional file 1: Figs. S2, S3). The frequency of corner visits was similar in both genotypes (Fig. 7A), but Avil-ChR2 mice made more nosepokes (NP) per visit (Fig. 7B) indicating more intensive exploration or compulsive behavior. In agreement with compulsiveness, lickings were increased particularly in free adaptation (FA) where licking times were unrestricted (Fig. 7C, Additional file 1: Fig. S3). In the other tasks, doors automatically closed after 5 s. Increased hedonic licking has indeed been observed in studies of chronic pain in mice [29, 64]. The ratios of NP-per-Visit of Avil-ChR2 mice were particularly high during the initial easy place preference learning period (NP 3 corner) and the final PPL-reversal period. In this final PPL-reversal period, the proportion of correct corner visits was higher in Avil-ChR2 (Fig. 7D) associated with increased licking (Additional file 1: Figs. S3A, B, Fig. 8). In this task, the correct corner switched to a spontaneously preferred corner that had been excluded in NP3c. Hence, Avil-ChR2 mice had a seeming advantage and increased success in this final task owing to stronger adherence to habits.

Further parameters of learning including the steepness of the learning curves and number of trials needed to reach the criterion of success did not differ between genotypes, but Avil-ChR2 mice showed a high frequency of fast corner re-entries (Fig. 8A) that manifested as high “repetitiveness” (Fig. 8B), which is a Log-ratio of observed versus expected returns to a corner. Repetitiveness also manifested temporarily in a higher frequency of corner visits resulting in a higher density of the actograms (Additional file 1: Figure 2) and was associated with increased licking behavior including licks per hour and licking duration (Fig. 8C–E). Repetitiveness, and licking parameters differed significantly between groups (Fig. 8C, D, Additional file 3: Excel Tables). In agreement, Random Forest feature selection revealed that repetitiveness and licking behavior accounted for the strongest differences between genotypes (Fig. 8E, Additional file 3: Tables Excel). Overall IC observations show an increased NP-per-visit ratio, repetitiveness and compulsive licking of Avil-ChR2 mice without impairment of learning and memory.

### Reduced brain and plasma sphingomyelins in Avil-ChR2 mice

**Fig. 9:**
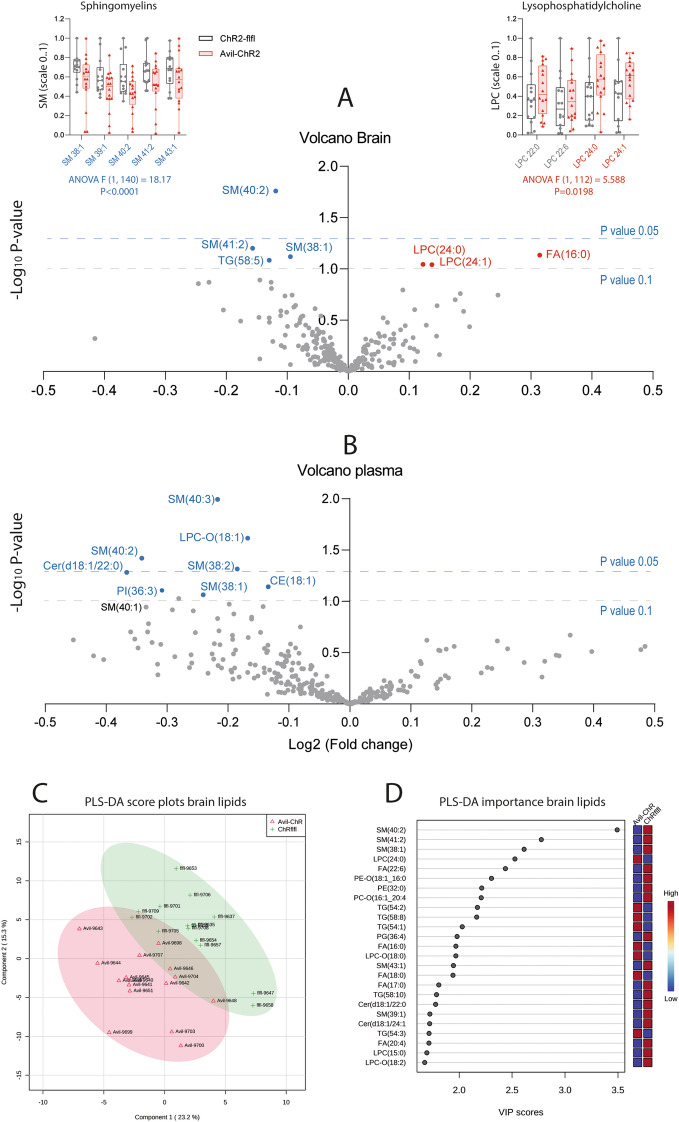
Lipidomic analyses show reduced sphingomyelin species in Avil-ChR2 brain and plasma. **A** Volcano plot of the log2 difference (fold change) of lipids in the brain (quantified as AUC/IS) versus the negative logarithm of the t-test P-value. Prominent spots are labelled with the lipid name. Blue lipids were reduced in Avil-ChR2 mice, red lipids were increased. The data are of n = 15 ChR2-flfl and n = 16 Avil-ChR2 female mice. The inserts show scatter plots of regulated sphingomyelins (SM; reduced, blue) and lysophosphatidylcholine (LPC(24:0), LPC(24:1)); red, increased). For scatter plots, lipids were normalized to range 0.1. **B** In analogy to A the Volcano plots show lipids in plasma (quantified as AUC/IS). Blue labeled lipids were reduced in Avil-ChR2 mice. **C**, **D** Score plot and variable importance (VIP) plot of Partial Least Square (PLS-DA) analysis of brain lipids. The features’ importance agrees with the ranking according to t-test P values

We hypothesized that subtle behavioral features may manifest in, or originate from, alterations of brain metabolism, particularly of ceramides and sphingomyelins (SM) because they have been implicated in mood disorders such as depression, anxiety, compulsiveness and addiction [40, 65–67]. We therefore performed brain and plasma lipidomic studies at the end of the behavioral observations. Volcano plots in Fig. 9A, B show reduced levels of long-chain sphingomyelin species, mainly SM of 38 and 40 C-atoms in brain and in plasma. At both sites, SM(40:2) was reduced. The inserts in Fig. 9A show individual SM species in individual mice. ANOVA confirmed significantly lower levels in Avil-ChR2 mice. The respective plot for plasma is shown in Additional file 1: Figure S4. Discriminant partial least square analysis (PLS-DA) (Fig. 9C, D) was used to reduce the dimensionality of data and extract key features. Scatter plots of PLS-1 versus PLS-2 separated genotypes with some overlap of the 95% confidence ellipses. The variable importance plot (Fig. 9E) again shows that SM 38, 40 and 41 are the top features that explain the difference between groups. XY-scatters of SM(40:2) in the brain versus SM(40:3) in plasma show a clear separation of the genotypes similar to PLS scores, showing that these SM are the important candidates (Additional file 1: Figure S4D). The data reveal changes of sphingomyelin metabolism in the brain of Avil-ChR2 mice which is reflected by similar changes in plasma. In conjunction with previous reports, the observed lipid patterns would agree with low sphingomyelin synthase activity or with enhanced sphingomyelin degradation.

### Increased calcium influx of TRP channels in primary DRG neurons

**Fig. 10:**
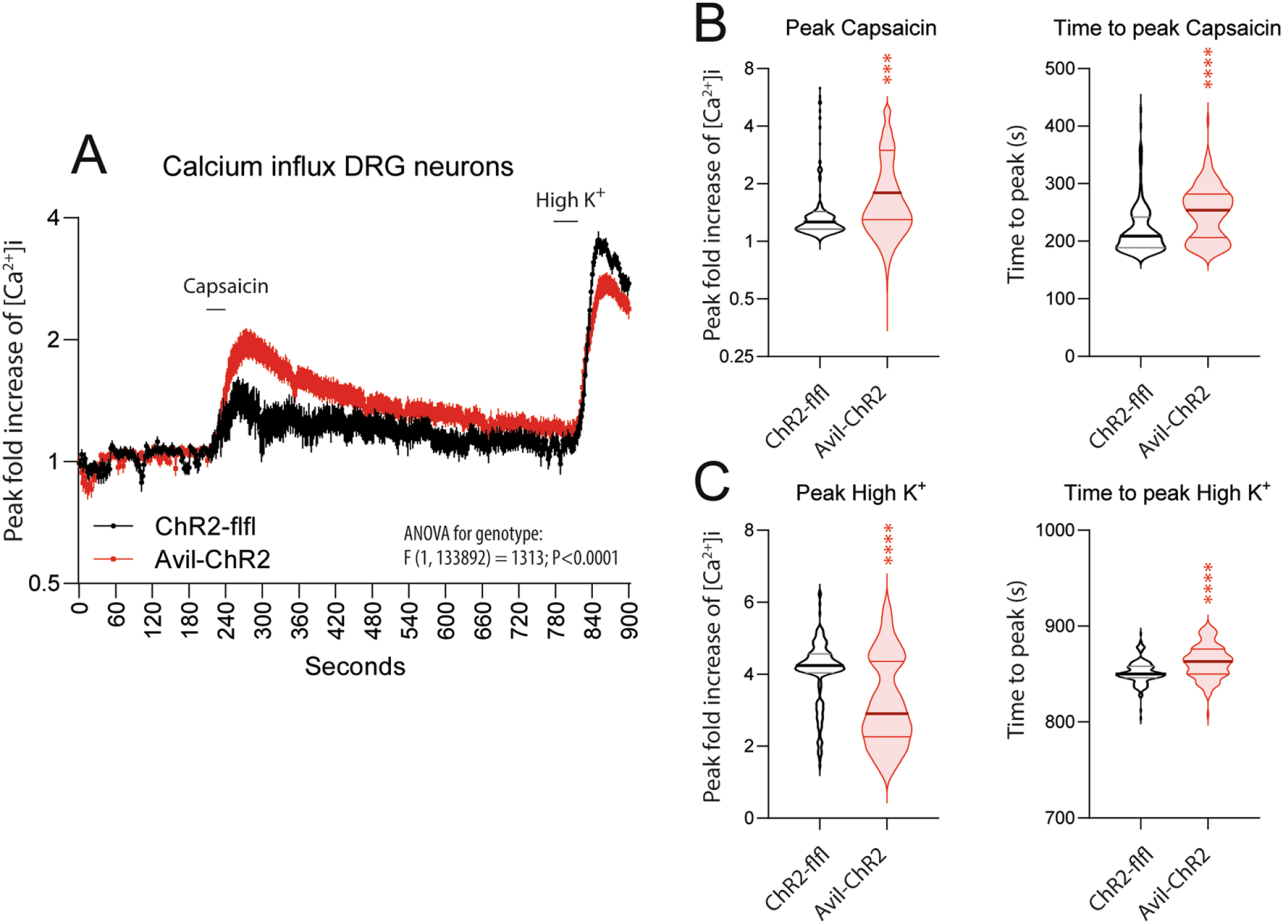
Capsaicin and high K^+^ evoked calcium influx in primary DRG neurons of ELP mice. ChR2-flfl and Avil-ChR2 mice were exposed to blue light in a chamber on postnatal day P1-P5 together with the Cre-negative blue-insensitive mother. DRGs were obtained for calcium imaging experiments of n = 4 female mice per genotype at 50 weeks of age after finishing behavioral studies. **A** Time course of the calcium influx at baseline (0–200 s) and on stimulation with 0.1 µM capsaicin (100–200 s) to stimulate TRPVs positive DRG neurons and subsequently with high K^+^ (50 mM KCl) for 45 s (780–825 s) to evoke depolarization-evoked calcium influx. Data are presented as changes in fluorescence ratios (F340/380) normalized to baseline ratios and show means ± 95% confidence intervals CI, n = 150 neurons per genotype. **B** Violin plots of the capsaicin peak ratios and the ‘time to peak’. The peak fold increase was obtained by integration and was the first peak of at least 5 consecutive ratios greater than 10% above baseline. The line shows the median, the dotted lines show the interquartile range. The violin shows the distribution, obtained by Kernel density estimation. **C** In analogy to **B**, violin plots show the peak fold increase of [Ca^2+^]_i_ upon stimulation with high K^+^ perfusion. This was defined as the peak and time to peak > 780 s with greater than 20% raise above baseline of a minimum of 5 consecutive data points. The peak increase [Ca.^2+^]_i_ and the time to peak were compared with 2-sided, unpaired t-tests. Time courses were compared by 2-way ANOVA. Asterisks indicate statistically significant differences, ***P < 0.001; ****P < 0.0001

Finally, we asked if nociceptive hypersensitivity was still evident as capsaicin hyperexcitability of primary DRG neurons at one year of age. Capsaicin responses of primary nociceptive neurons are a biological correlate of peripheral pain hypersensitivity and are mediated through TRP channels including TRPV1 and TRPV4 [68, 69]. High-K^+^ was used to assess depolarization evoked calcium currents and neuron viability. Baseline 340/380 nm ratios were similar, but capsaicin evoked calcium influx was stronger in neurons of Avil-ChR2 mice as compared to control neurons of ChR2-flfl mice. Inversely, high K^+^ evoked peak calcium influx was lower in Avil-ChR2 neurons likely because neurons were still refractory (Fig. 10). The proportion of K^+^ responding neurons were similar in both groups (90.2% in ChR2-flfl; 95.5% Avil-ChR2). The results suggest that ELP evoked nociceptive sensitization is maintained at a biological level and agrees with nociceptive hypersensitivity of aged Avil-ChR2 mice (Fig. 4B).

## Discussion

We show in the present study in an optogenetic mouse model that blue light evoked early life pain (ELP) causes cortical hyperexcitability and reduced expression of synaptic genes suggesting refinement of synaptic connectivity [61, 62, 70]. Post-ELP “psychopathology” in adult life manifested as nociceptive hypersensitivity and repetitive, compulsive behavior in the IntelliCage. Nociceptive hypersensitivity tended to normalize towards the end of the observation (one year) but DRG neurons of aged mice were still hypersensitive upon stimulation with capsaicin, and behavioral features were associated with low long-chain sphingomyelin species in brain and plasma pointing to abnormal activity of sphingomyelin metabolism which has be suggested a key mechanism in psychiatric disorders [40, 42, 43, 71, 72]. The data are a strong argument for measures against ELP.

It is of note that Avil-ChR2 mice were not impaired in daily mouse life. They behaved comparable to controls in classical maze tests of anxiety, curiosity, spatial cognition, social cognition and memory. IntelliCage behavioral parameters of activity, circadian rhythms, social structure, and learning & memory were also equal to controls. Hence, differences between genotypes were more subtle. Avil-ChR2 mice showed a high rate of nosepokes per visit and high frequency of repetitive returns to the same corner irrespective of the success within this corner, plus compulsive licking behavior. One may interpret this behavior as compulsive repetitiveness i.e. insistence on sameness and cognitive inflexibility [73–75] considered as features of reward deficiency syndrome [76, 77]. One would expect difficulties in reversal learning [78–80], but accuracy in preference learning or reversal learning was not affected. We even noticed a paradoxical higher accuracy in the final reversal period likely reflecting strong spontaneous habits. IntelliCage designs strive to minimize biases of spontaneous preferences, but it cannot be completely avoided. In this final task, reward was assigned to one corner, which had been highly preferred during adaptation. This was true for all mice, but obviously Avil-ChR2 mice adhered more strongly.

An alternative explanation for outperformance of Avil-ChR2 is suggested by a study that revealed a paradoxical enhancement of reversal learning under mild stress [81], which would imply that ELP mice were under mild stress. In support of this hypothesis, adolescent to young adult mice had temporarily lower body weights as compared to controls, and aged Avil-ChR2 mice engaged less in rewarding voluntary running. The performance in motor function tests was normal. Therefore, low VWR indeed points to lower reward. ELP might have reduced suckling, so that weaning body weights were lower than in controls but they caught up with the controls' body weights and were as healthy as the controls. During the IntelliCage experiments, body weights were equal in both groups, and we did not observe social structures suggestive of inferiority of Avil-ChR2 mice. Still, it is possible from a human perspective that ELP might cause persistent mild stress in adulthood.

Previous studies have employed repeated pin prick [18], skin incision [16, 19, 21, 82] or nerve injury [6] of neonatal mice or rats to assess the impact of ELP. The results mostly show that such neonatal injuries increase nociceptive sensitivity in adult life and cause a more serious course of a second injury in adult life [7, 20, 21]. The resulting neuroimmune activation [6, 7] and low endocannabinoid tone [5] may predispose to metabolic disease or reward deficiency syndromes [77]. In our optogenetic ELP model we did not observe an immune activation (RNAseq) but stimulated nociceptive withdrawal thresholds were lower than in controls, showing nociceptive sensitization. There was no evidence for spontaneous heat or cold intolerances as assessed with a TGR, in which mice can freely choose the preferred temperature zone [44, 83]. Avil-ChR2 mice were equal to controls in the TGR. Hence, the sensation of warmth and cold was not affected. In particular, the settings would have revealed cold intolerance [83, 84]. Importantly, normal TGR behavior shows that blue light evoked excitation of ChR2 in neonatal Avil-ChR2 mice did not damage sensory neurons or skin that would have manifested in some kind of sensory neuropathy and loss of thermal sensation [85]. The viability of primary DRG neurons as assessed as proportion of high K^+^ responsive neurons did not differ between genotypes. Instead, capsaicin evoked calcium influx was stronger in DRG neurons of Avil-ChR2 mice suggesting hypersensitivity or increased expression of transient receptor potential TRPV1 channels, which is a biological correlate of heat pain [86, 87]. Calcium imaging results thus agree with the behavioral tests of nociception and reveal the peripheral nociceptive sensitization at a biological level.

Pain sensitization may arise in the periphery at the level of the primary sensory nerve or nerve terminal [88, 89] and/or may involve hyperexcitability of the central nociceptive system [90], referred to as “pain matrix” in functional magnet resonance imaging (fMRI) studies [91–93]. We used electrophysiological MEAchip recordings from cortical slice preparations to address the central sensitization evoked by ELP. These multi-electrode recordings were done after completion of blue light stimulation with a free interval of 3–4 days. Hence, the observed higher frequency of spontaneous action potentials suggests that ELP elicits cortical hyperactivity that outlasts sensory stimulations. Owing to our mouse model of *Advillin*-driven [31, 33] ChR2 expression primarily in IB4 positive nociceptors during the stimulation period, we assume that blue light penetrated the skin to activate sensory nerve terminals [29, 36] but did not directly activate cortical neurons, also prevented by the skull.

It has been shown previously that somatosensory touch or whisker evoked stimulation of the cortex in the early days of life leads to an increased rate of neuronal apoptosis by P7 hence matching the time of our transcriptomic and electrophysiology studies [9, 10, 94]. We did not observe differences between genotypes of active caspase 3 immunofluorescence or neurogenesis or activation of apoptosis associated genes, but RNAseq showed a reduced expression of synaptic genes in Avil-ChR2 mice including *Grin2b*, neurexins, *piccolo* and voltage gated calcium and sodium channel subunits. Transcriptomic changes would agree with fewer synaptic contacts possibly owing to a refinement of neuronal networks that were highly in use upon nociceptive stimulation [9]. In the context of early life injuries such as skin incision, such priming was shown to increase the response to injuries in later life [20, 21], a phenomenon that is believed to involve central sensitization and immune activation. It is important to note, that our mice had no skin or tissue injury, and transcriptomics reveal that blue light exposure did not elicit neuroinflammation that was described in neonatal injury models [6, 7].

We have shown previously using adult Avil-ChR2 mice that blue light evokes paw withdrawal and active avoidance in a chamber [29]. Hence, we assume that blue light elicits an unpleasant feeling interpreted as “pain”. Originally, advillin expression was proposed to occur in all somatosensory neurons which was based on studies in embryonic mice [31, 32]. However, later, a very detailed analysis in postnatal mice revealed that advillin is enriched in IB4 positive non-peptidergic nociceptors in postnatal DRGs and not equally expressed in all DRG neurons [34], also confirmed in a study in adult mice [35]. Hence, blue light stimulation in our mice likely mostly activated non-peptidergic nociceptors which agrees with blue light avoidance in adult mice. Nevertheless, it cannot be excluded that blue light also activated some Merkel cells in the skin, which were shown to express advillin [34]. Merkel cells are mainly found in glabrous skin of the paws and involved in sense of pressure. Advillin expression also occurs in autonomous nerves and ganglia, but expression in the autonomous nervous system only emerges beyond P7, i.e. when the blue light stimulation was already finished. Hence, considering advillin expression after birth and the stimulation protocol in our study, we believe that blue light mildly activated non-peptidergic nociceptors and was unpleasant but not harmful. This notion is supported by the observed high spontaneous cortical firing activity that outlasted sensory stimulation, and is reminiscent of cortical hyperactivity after nerve injury [95], neuroinflammation [96] or traumatic injury [97, 98]. Different from early life stress models imposed by intermittent maternal deprivation [77, 99–101], perinatal immune activation [102, 103] or perinatal valproic acid treatment [74, 104, 105] our mice did not show behavior of autism like social deficits, depression or anxiety or features of schizophrenia or cognitive impairment. Indeed, Avil-ChR2 mice behaved astonishingly equal to controls in all standard maze tests showing that the blue light exposure was not harmful. Differences revealed only upon detailed analyses of IntelliCage behavior. Key parameters that were consistently altered in successive tasks were the frequency of rapid returns to the same corner, quantified as “repetitiveness”, and the numbers and durations of lickings. In addition, Avil-ChR2 made more nosepokes per visit (NP-per Visit ratio) in some tasks. NP/visit ratios are normally high in young mice showing strong exploration and are low in mice with dementia-associated hyperactivity [57, 106]. Together with the repetitiveness and “over-licking”, high NP/visit ratios of Avil-ChR2 mice suggest compulsive behavior and insistence on sameness rather than youthful exploration. From a human perspective, one is inclined to interpret the behavior of Avil-ChR2 as subtle but still important psychopathology which might be a consequence of persistent nociceptive hypersensitivity or develop independently in consequence of ELP.

At the biological level, psychopathology of ELP mice was associated with low brain and plasma levels of sphingomyelin species, pointing to alterations of sphingomyelin metabolism, that have been suggested to contribute to neuropsychiatric diseases, but mechanistically, the pathology is still poorly understood [43]. It has been shown that the activity of sphingomyelin degrading enzymes, neutral or acidic sphingomyelinase, is increased in psychiatric diseases including depression, anxiety and addiction [40, 42, 43, 71]. Some antidepressants work as functional inhibitors of acidic sphingomyelinase. It is therefore believed that raising SM is part of their mood stabilizing effects [72, 107]. Our results show low SM 38, 40 and 41 species in brain and plasma. The characterization of SM species is a recent advancement in lipidomic analyses and it is not known yet how specific SM species work in the context of psychic health. Based on previous reports and our results it is tempting to speculate that the late ELP-psychopathology of Avil-ChR2 mice is caused / contributed by changes of sphingomyelin homeostasis.

### Supplementary Information

**Additional file 1.** Supplementary figures and legends

**Additional file 2. **Normalized RNAseq reads of candidate genes

**Additional file 3.** Supplementary statistical analyses of IntelliCage behavior

**Additional file 4.** 3D brain ChR2-flfl control mouse, active Caspase 3, Light-Sheet-Microscopy

**Additional file 5.** 3D brain Avil-ChR2 mouse, active Caspase 3, Light-Sheet-Microscopy

## Data Availability

RNA sequencing data have been deposited to the GEO database with the accession number GSE200140.
