# Supplementary material for: Repetitive and compulsive behavior after Early-Life-Pain associated with reduced long-chain sphingolipid species
Source: Cell Biosci. 2023 Aug 27;13:155. doi: 10.1186/s13578-023-01106-3 (PMC10463951; doi:10.1186/s13578-023-01106-3)
Supplement: Supplementary file 1 — Additional file 1. Supplementary figures and legends [file 13578_2023_1106_MOESM1_ESM.docx]

# Additional to:

Repetitive and compulsive behavior after Early-Life-Pain associated with reduced long-chain sphingolipid species

Alexandra Vogel^1^, Timo Ueberbach^2^, Annett Wilken-Schmitz^1^, Lisa Hahnefeld^1,3,4^, , Luisa Franck^1^, Marc-Philipp Weyer^1^, Tassilo Jungenitz^5^, Tobias Schmid^6,7^, Giulia Buchmann^8^, Florian Freudenberg^9^, Ralf P. Brandes^8^, Robert Gurke^1,3,4^, Stephan W. Schwarzacher^5^, Thomas Mittmann^2^, Irmgard Tegeder^1^

**Abstract**

**Background:** Pain in early life may impact on development and risk of chronic pain. We developed an optogenetic Cre/loxP mouse model of "early-life-pain" (ELP) using mice with transgenic expression of channelrhodopsin-2 (ChR2) under control of the *Advillin* (*Avil*) promoter, which drives expression of transgenes predominantly in isolectin B4 positive non-peptidergic nociceptors in postnatal mice. Avil-ChR2 (Cre+) and ChR2-flfl control mice were exposed to blue light in a chamber once daily from P1-P5 together with their Cre-negative mother. **Results:** ELP caused cortical hyperexcitability at P8-9 as assessed via multi-electrode array recordings that coincided with reduced expression of synaptic genes (RNAseq) including *Grin2b*, neurexins, *piccolo* and voltage gated calcium and sodium channels.. Young adult (8-16 wks) Avil-ChR2 mice presented with nociceptive hypersensitivity upon heat or mechanical stimulation, which did not resolve up until one year of age. The persistent hypersensitivy to nociceptive stimuli was reflected by increased calcium fluxes in primary sensory neurons of aged mice (1 year) upon capsaicin stimulation. Avil-ChR2 mice behaved like controls in maze tests of anxiety, social interaction, and spatial memory but IntelliCage behavioral studies revealed repetitive nosepokes and corner visits and compulsive lickings. Compulsiveness at the behavioral level was associated with a reduction of sphingomyelin species in brain and plasma lipidomic studies. Behavioral studies were done with female mice. **Conclusion:** The results suggest that ELP may predispose to chronic "pain" and compulsive psychopathology in part mediated by alterations of sphingolipid metabolism, which have been previously described in the context of addiction and psychiatric diseases.


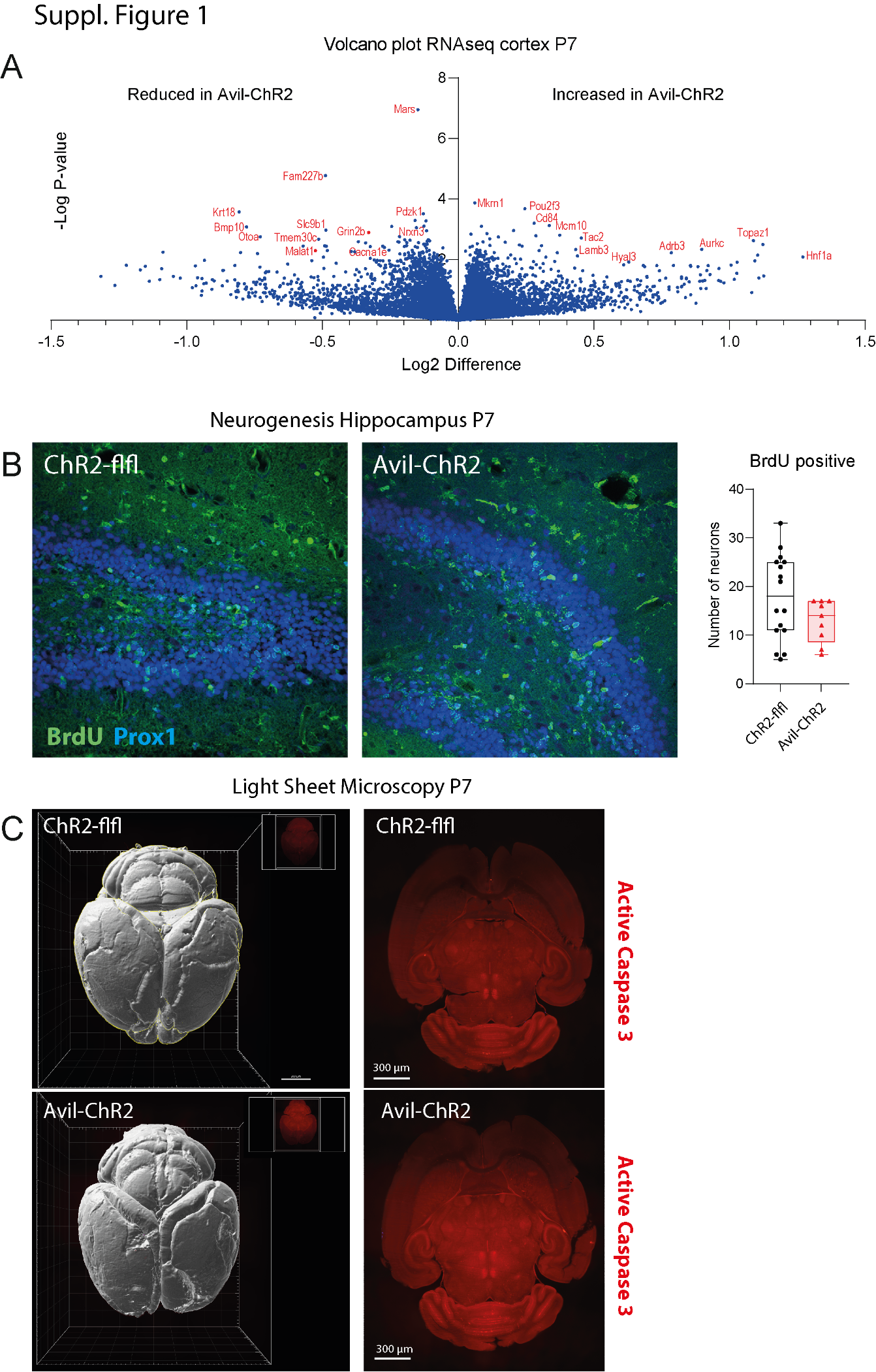


# Figure S1

### RNA sequencing of the brain cortex, BrdU neurogenesis and active Caspase 3 in Early-Life-Pain mice at P7

ChR2-flfl and Avil-ChR2 mice were exposed to blue light in a chamber on postnatal day P1-P5 together with the Cre-negative blue-insensitive mother. At P7, mice were euthanized, and brain cortices subjected to transcriptome analysis via mRNA sequencing or morphologic studies of hippocampal neurogenesis (B) and 3D imaging of active caspase 3 (C).

**A:** Volcano plot of the log2 difference (fold change) versus the negative logarithm of the t-test P-value. Prominent spots are labelled with the gene name in red. (n = 8 pups per genotype)

**B:** For analysis of neurogenesis, BrdU was injected i.p. on P1, P3 and P5 for fluorescent labeling of newborn neurons. Mice were euthanized at P7 and were transcardially perfused with saline followed by PFA for fixation. BrdU (green) immunofluorescence of newborn neurons and Prox1 (blue) granule cell specific counterstaining in ChR2-flfl and Avil-ChR2 P7 mice. Images of the hippocampus show representative results of n = 6 mice per genotype. BrdU positive neurons were marked and counted manually in FIJI ImageJ. The numbers did not differ between genotypes (unpaired, 2-tailed t-test). The boxes show the interquartile range, the line is the median, whiskers show minimum to maximum, and the scatters show 1-3 images of n = 6 mice.

**C:** For Light Sheet microscopy and 3D reconstruction PFA fixated P7 brains were subjected to tissue iDISCO+ tissue clearing and subsequent immunostaining of active caspase-3 to reveal apoptotic neurons. The images show the 3D brain reconstruction and a section of one exemplary mouse per genotype of n = 4 mice per genotype. Two exemplary supplementary mp4 video files allow scanning through the brain. Brain morphology did not differ between genotypes.


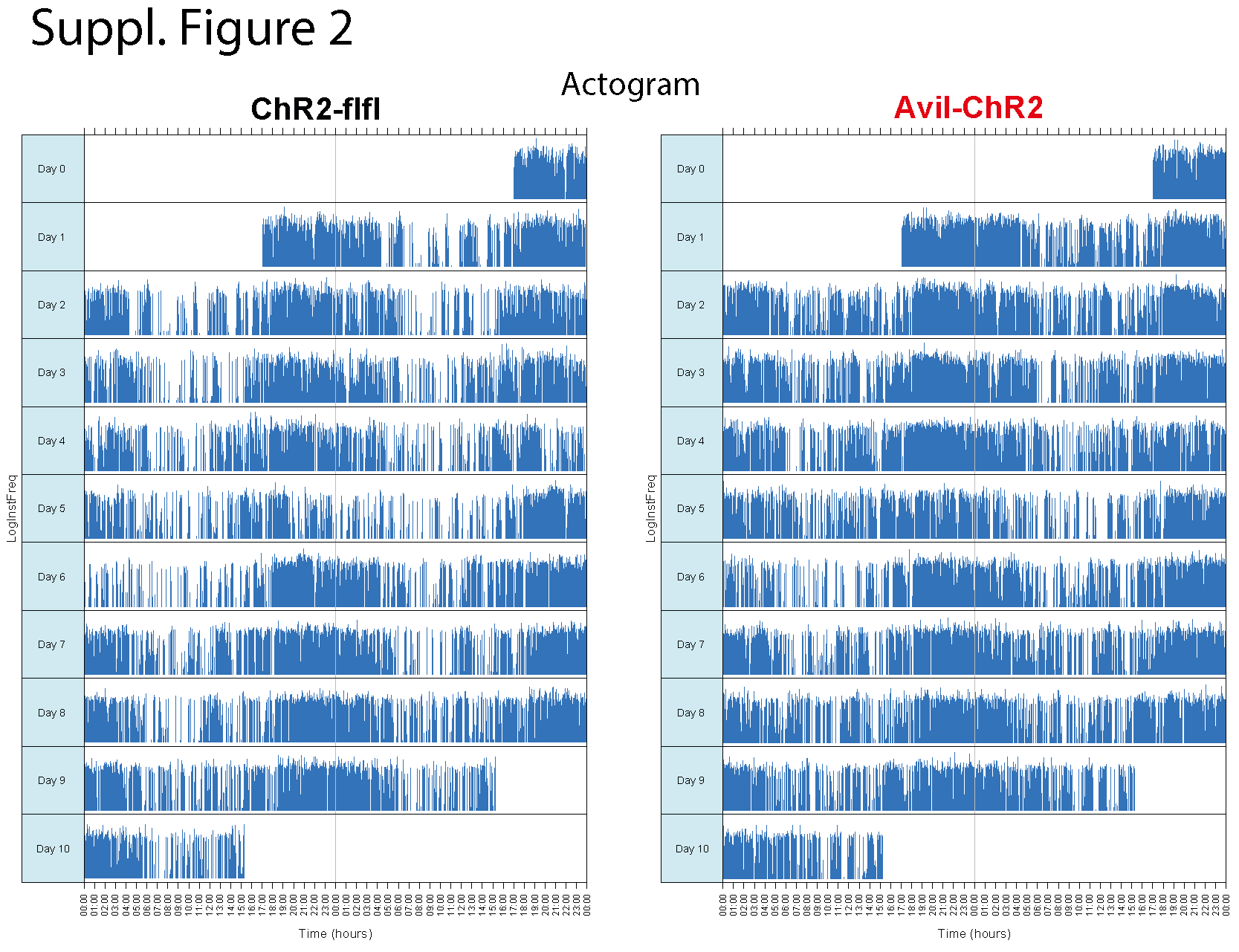


# Figure S2

Actogram of ChR2-flfl (n = 15; left) and Avil-ChR2 mice (n = 16; right) during Place Preference Learning in the IntelliCages. Log2 frequencies of corner visits are plotted per hour relative to the time of Light Off. Circadian rhythms are alike, but activity is higher in Avil-ChR2 mice (higher density of blue spikes) particularly during daytime.


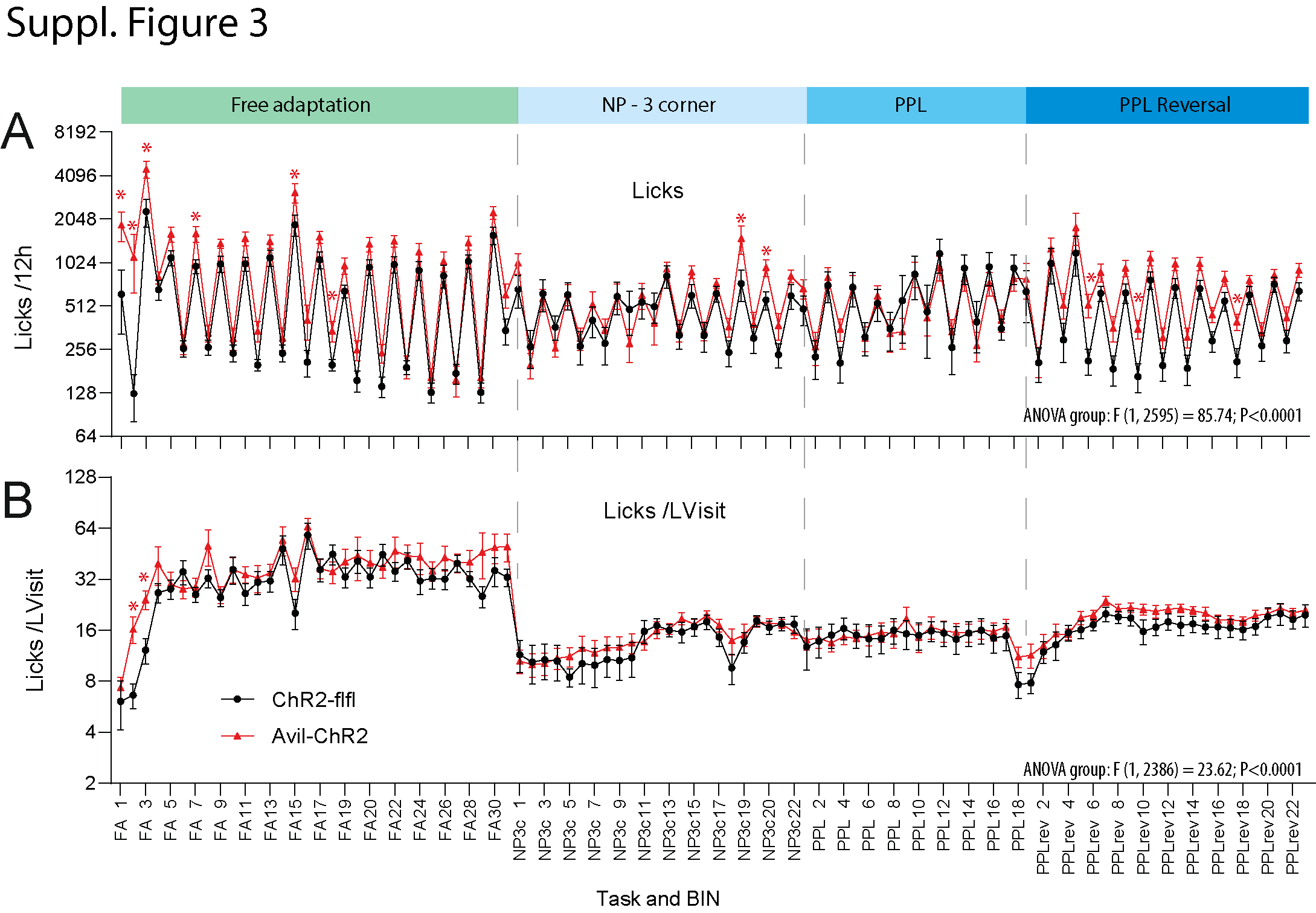


# Figure S3

### IntelliCage licking behavior

ChR2-flfl and Avil-ChR2 mice were exposed to blue light in a chamber on postnatal day P1-P5 together with the Cre-negative blue-insensitive mother. IntelliCage observations started at 30 weeks of age and lasted up to 18 weeks. The experiment included n = 15 ChR2-flfl and n = 16 Avil-ChR2 female mice. Mice were trained in sequential tasks of increasing difficulty. Tasks were free adaptation (FA), nosepoke adaptation (NP and NP 3 corner, NP3c), place preference learning (PPL) and reversal place preference learning (PPL-rev).

**A:** Time course of the number of licks in 12h Bins in sequential tasks. The fluctuations of licking activity reveal the circadian rhythms.

**B:** Time course of the frequency of licks per visit with licks (Licks /LVisit) in sequential IntelliCage tasks. Licks/Visits depend on the tasks, the ratio is high during free adaptations (all doors are open, no restriction) but drop and remain constant in nosepoke adaptations and preference learning tasks.

Time courses were compared by 2-way ANOVA with the within subject factor "time" and the between subject factor "genotype" and subsequent posthoc analysis for genotype. ANOVAs differed between genotypes. Licking behavior was higher in Avil-ChR2 mice at the onset of IC experiments in FA. Minor differences in PPL-reversal were not significant.


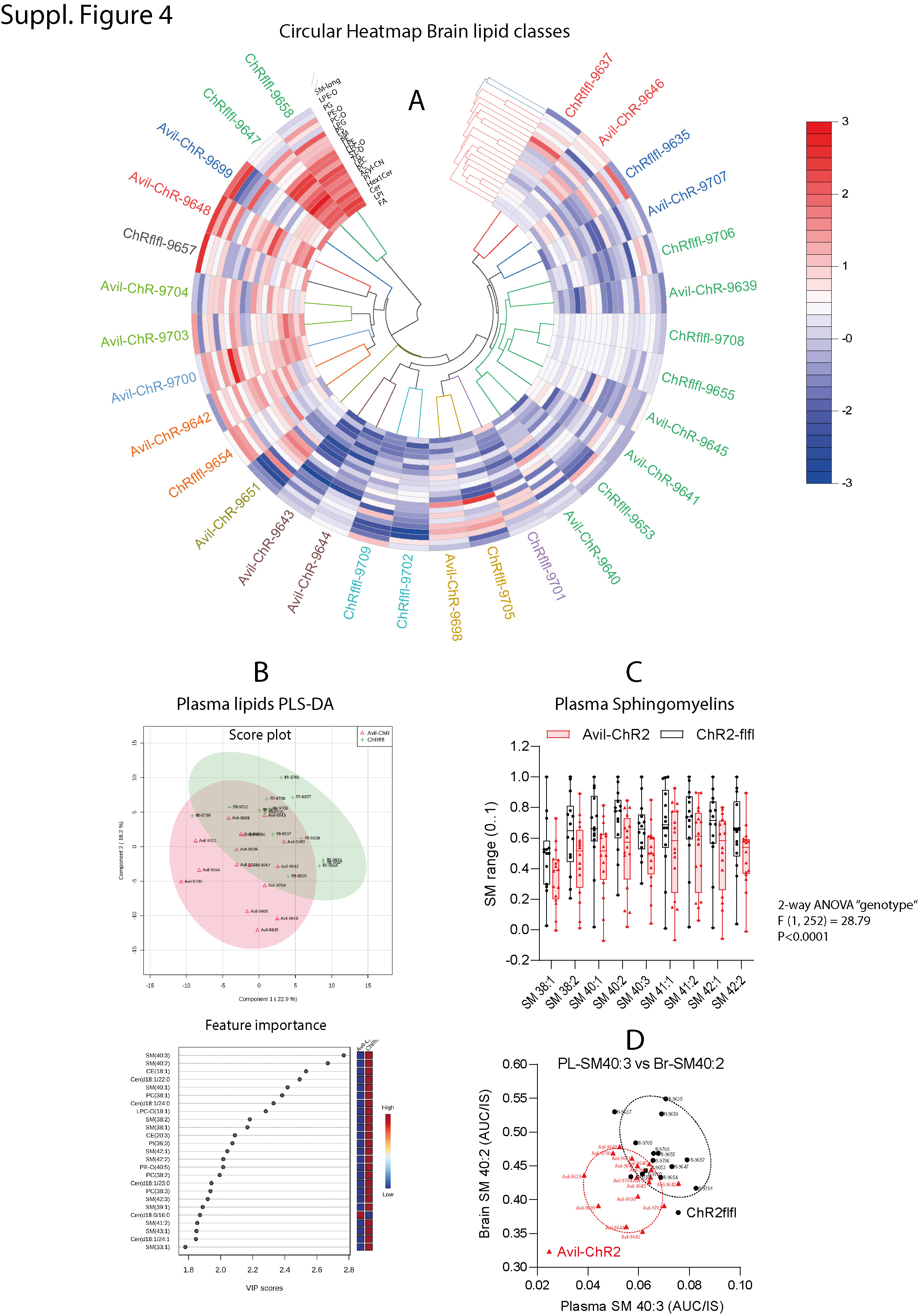


# Figure S4

A: Circular heatmap with dendrogram of brain lipid classes with clustering of lipid classes (rows) and mice (radial). Mice clustered in two major groups, but not according to genotypes.

B: Score plot and variable importance (VIP) plot of Partial Least Square (PLS-DA) analysis of plasma lipids. The features' importance agrees with the ranking according to t-test P values.

C: Box/Scatter plots show regulated sphingomyelins in plasma. For scatter plots, lipids were normalized to range 0..1. Each scatter represents one mouse, n = 15 for ChR2-flfl and n = 16 for Avil-ChR2 mice.

D: XY-Scatter plot of SM 40:3 in plasma versus SM 40:2 in brain tissue of n = 15 for ChR2-flfl and n = 16 for Avil-ChR2 mice. Sphingomyelins with 40 C-atoms were among the top regulated SMs. The scatter clouds of ChR2-flfl and Avil-ChR2 mice overlap, but agree with PLS-DA score plots, suggesting that these sphingomyelins are key features that differ between genotypes.

# Additional list of internal standards of lipidomic analyses

Working solution with internal standards for lipidomic analysis (75 µl IS solution for 10 µl of sample)

| **Internal Standard** | **Concentration (µg/ml)** |
| --- | --- |
| Arachidonic acid d8 | 0.1 |
| CE 18:1-d7 | 5 |
| Cer d18:1/16:0-d7 | 0.02 |
| Cholesterol-d7 | 7.5 |
| DG 15:0/18:1-d7 | 0.3 |
| LacCer d18:1/17:0 | 0.06 |
| LPC 18:1-d7 | 0.3 |
| LPC O-16:0-d4 | 0.02 |
| LPE 18:1-d7 | 0.02 |
| LPG 17:1 | 0.02 |
| LPI 17:1 | 0.02 |
| PC 15:0/18:1-d7 | 2 |
| PC O-18:0/18:1-d9 | 0.2 |
| PE 15:0/18:1-d7 | 0.1 |
| PE O-18:0/18:1-d9 | 0.1 |
| PG 15:0/18:1-d7 | 0.1 |
| PI 15:0/18:1-d7 | 0.1 |
| PS 15:0/18:1-d7 | 0.025 |
| SM d18:1/18:1-d9 | 0.4 |
| TG 14:0/16:1/14:0-d5 | 0.6 |
| TG 15:0/18:1-d7/15:0 | 0.6 |
| TG 20:0/20:1/20:0-d5 | 0.6 |
